# Exploring the Critical Components and Therapeutic Mechanisms of *Perilla frutescens L.* in the Treatment of Chronic Kidney Disease via Network Pharmacology

**DOI:** 10.3389/fphar.2021.717744

**Published:** 2021-11-26

**Authors:** Chen Yong, Zhengchun Zhang, Guoshun Huang, Yang Yang, Yiye Zhu, Leilei Qian, Fang Tian, Li Liu, Qijing Wu, Zhongchi Xu, Chong Chen, Jing Zhao, Kun Gao, Enchao Zhou

**Affiliations:** ^1^ Division of Nephrology, Affiliated Hospital of Nanjing University of Chinese Medicine, Jiangsu Province Hospital of Chinese Medicine, Nanjing, China; ^2^ Division of Nephrology, JiangYan Hospital affiliated to Nanjing University of Chinese Medicine, Taizhou, China; ^3^ Division of Nephrology, The People’s Hospital of Rugao, Rugao, China; ^4^ Research Center of Chinese Medicine, Jiangsu Province Hospital of Chinese Medicine, Nanjing, China

**Keywords:** Perilla frutescens L., chronic kidney disease, network pharmacology, luteolin, critical component, therapeutic mechanism

## Abstract

Chronic kidney disease (CKD) is a chronic progressive disease that seriously threatens human health. Some patients will continue to progress into the CKD stage 3–5 (also called chronic renal failure), which is mainly manifested by a decline in renal function and multi-system damage. *Perilla frutescens (L.) Britton. (Lamiaceae)* is one of the most widely used traditional Chinese medicine (TCM) herbs in CKD, especially in CKD stage 3–5. But its active components and mechanisms are still unclear. In this study, we used network pharmacology to analyze the active components of *P. frutescens* and the main therapeutic targets for intervention in CKD stage 3–5. Then, the key components were selected for enrichment analysis and identified by high performance liquid chromatograph (HPLC). Finally, we verified the critical components through molecular docking, and *in vitro* experiments. The results show that 19 main active components of *P. frutescens* were screened, and 108 targets were intersected with CKD stage 3–5. The PPI network was constructed and found that the core nodes AKT1, TP53, IL6, TNF, and MAPK1 may be key therapeutic targets. Enrichment analysis shows that related targets may be involved in regulating various biological functions, and play a therapeutic role in CKD stage 3–5 by regulating apoptosis, T cell receptor, and PI3K-AKT signaling pathways. Molecular docking indicates that the key active components were well docked with its corresponding targets. Five active components were identified and quantified by HPLC. According to the results, luteolin was selected as the critical component for further verification. *In vitro* experiments have shown that luteolin can effectively alleviate adriamycin (ADR)-induced renal tubular apoptosis and suppress AKT and p53 phosphorylation. The effects of luteolin to reduce apoptosis may be mediated by inhibiting oxidative stress and downregulating the mitogen-activated protein kinase (MAPK) and p53 pathways. In general, we screened and analyzed the possible active components, therapeutic targets and pathways of *P. frutescens* for treating CKD. Our findings revealed that luteolin can reduce renal tubular epithelial cell apoptosis and may be the critical component of *P. frutescens* in the treatment of CKD. It provides references and direction for further research.

## Introduction

Chronic kidney disease (CKD) stage 3–5 is traditionally referred to as chronic renal failure (CRF). CKD is a chronically progressive clinical syndrome that mainly manifests as kidney damage and can involve multiple organs throughout the body. It is the common end stage of various kidney diseases. Various factors lead to decompensation of renal function, resulting in the retention of water, electrolyte and metabolites, acid-base balance disorder, and other systemic injuries. CKD is characterized by a long course, complicated conditions, difficult treatment, and poor prognosis. It seriously affects the quality of life and health, and brings a heavy economic and health burden to the family and society (Qiu et al., 2017). There is an urgent need to find new and effective therapeutic strategies to slow the progression of CKD.

Traditional Chinese medicine (TCM) provides an alternative treatment for CKD. Several studies have demonstrated the safety and efficacy of TCM in CKD (Song et al., 2019; Xi et al., 2020). The use of TCM in nephrology has attracted interest. Herbs are an important part of TCM intervention. Some herbs can effectively delay the progression of CKD and reduce the incidence of complications through synergistic effects with a variety of pharmacodynamic substances. A series of studies have addressed the value of TCM herbs in CKD therapy. The delayed progression of CKD that has been described mainly involves reduced oxidative stress, inhibition of the inflammatory response, reduced renal cell apoptosis, and alleviation of renal fibrosis (Lu et al., 2018; Wang et al., 2018; Fu and Hu, 2019; Zhao et al., 2019). In addition, there is a mutual crosstalk among these pharmacological effects, which reflects the multi-target effects of TCM herbs.

The leaf of *Perilla frutescens (L.) Britton. (Lamiaceae)* is used to treat patients with CKD (especially stage 3–5) in the clinic (Guangtang and Huiman, 2017). The treatment can significantly delay the progression of CKD. In TCM theory, it can disperse the poison in the blood and harmonize the digestive system. Our team previously confirmed that a TCM formulation composed mainly of *P. frutescens* can significantly improve renal function and increase the estimated glomerular filtration rate (Yang and Enchao, 2015). *In vitro* and *in vivo* experiments have confirmed that it can alleviate the oxidative damage of renal tubular epithelial cells, reduce renal mesangial cell dysfunction, and delay renal fibrosis (Yiye et al., 2019; Kim and Kim, 2019). However, the active components of *P. frutescens* and details of its effects for treating CKD are still unclear.

Network pharmacology refers to the use of computer simulations and high-throughput analytical methods to reveal complex pharmacological behaviors (Xinqiang et al., 2020). In TCM research, the process mainly includes identifying related genes of the disease treated by TCM herbs and constructing a network model to evaluate the influence of TCM herbs on disease networks. Especially concerning TCM intervention in chronic and complex diseases, such as CKD, it is helpful to clarify the mechanism and confirm the specific targets and herbs. Molecular docking is a technology that screens active components through computer simulations of ligand binding to a target (Xinqiang et al., 2020). This technology is widely used in drug design and screening, as well as in structure-based virtual screening and ligand research. Combining the technology with network pharmacology can verify the analysis results in terms of the molecular structure.

In this study, we applied network pharmacology to systematically explore the main active components, targets and pharmacological mechanisms of *P. frutescens* for treating CKD. The binding ability of the key active components to the corresponding targets was analyzed by molecular docking. The screened components were quantified by HPLC and verified by *in vitro* experiments. So as to further identify the critical components of *P. frutescens* and evaluate its effects on kidney injury.

## Materials and Methods

### Collection and Screening of Main Active Components of *P. frutescens*

A search for all chemical components of *P. frutescens* was conducted in the traditional Chinese medicine systems pharmacology (TCMSP) database (http://lsp.nwu.edu.cn/index.php) using “zisu” as the key word. Filter criteria included database ADME (absorption, distribution, metabolism, excretion) parameters, select oral bioavailability (OB) ≥30%, and drug-likeness (DL) ≥0.18. In addition, the search focused on components that were effective in the relevant literature but did not meet the above screening criteria to supplement. Finally, the main active components of *P. frutescens* were identified.

### Target Prediction of the Main Active Components

The predicted targets of the main active components were retrieved from the TCMSP database. They were entered into the Uniport database (http://www.uniprot.org/) with the species restricted to “Human.” The official gene name of all target proteins was checked one-by-one. The genes constituted the predicted targets of the main active components.

### Collection of Targets Related to CKD

A search for target genes related to CKD stages 3–5 was performed in the Gene cards database (https://www.genecards.org/) with the keyword “chronic renal failure” (because CKD stages 3–5 are traditionally termed CRF). Due to the excessive number of CRF-related targets, a score ≥10 was used as the threshold to screen the main targets related to CKD stage 3–5 for further research.

### Acquiring Targets of Main Active Components for CKD Treatment

Venny 2.1.0 (http://bioinfogp.cnb.csic.es/tools/venny/index.html) software was used to obtain the intersection of the predicted targets of *P. frutescens* and the main targets of CKD stage 3–5. A Venn diagram was drawn. The intersection genes were possible relevant targets for the treatment of CKD with *P. frutescens*.

### Construction and Analysis of the Network Model of Main Active Components-CKD Treatment Related Targets

The main active components and the corresponding CKD stage 3–5 related targets were imported into Cytoscape3.6.0 software. The main active components-targets network model was drawn. Then, the Network Analyzer plugin was used to analyze the network topology and to calculate the degree, betweenness centrality, and closeness centrality parameter values. The larger the value, the more important the node is in the network, enabling the clarification of the core components and targets of *P. frutescens* in the treatment of CKD.

### Construction and Modular Analysis of Protein-Protein Interaction (PPI) Network Model

The core targets in the network model were imported into the String11.0 database (https://string-db.org/) with species limited to “homo sapiens.” The highest confidence protein score value was >0.7. Unconnected single proteins were removed to obtain the PPI diagram of these core targets. The relevant action information of these targets was imported into Cytoscape 3.6.0 software to construct a network model and perform topological analysis, and adjust the node size, color, and edge thickness according to the value of the degree and combined score. The degree value was used as a reference to filter out the key nodes in the PPI network and draw a bar graph. At the same time, the MCODE plugin for protein modular analysis was used to set Degree Cutoff = 2, Node Score Cutoff = 0.2, K-Core = 5, Max.Depth = 100, and to screen out key protein modules in the PPI network.

### Enrichment Analysis of Gene Ontology (GO) and Signaling Pathway

The relevant targets were imported into Cytoscape3.6.0 software. The ClueGO and CluePedia plugins were used to annotate the GO biological functions of these targets, including the enrichment of cellular components (CCs) and molecular functions (MFs). The analysis was set with a *p*-value ≤0.01 as the criterion to screen out the main biological functions of the core targets. The network model was established and a pie chart drawn.

Target information was imported into the DAVID6.8 online analysis platform (https://david.ncifcrf.gov/) for enrichment analysis of biological processes (BPs) and signaling pathways. The criterion and background were both “homo sapiens.” The GO-BP and Kyoto Encyclopedia of Genes and Genomes (KEGG) databases were used to enrichment analyze the BPs and pathways related to the targets. The smaller the *p*-value, the higher the degree of enrichment. Therefore, the main BPs and pathways of *P. frutescens* for CKD stage 3–5 treatment were selected using the criterion of *p* <0.01. The enrichment results were visualized by software R language 4.0.3 and clusterProfiler package.

### Molecular Docking Verification

The three-dimensional structure of the main active components of *P. frutescens* was downloaded from the PubChem database (https://pubchem.ncbi.nlm.nih.gov/) and OpenBabel software was used to convert it to the mol2 format. The complex crystal structure containing the original ligand of the key targets in the PPI network from the RSCB PDB database (http://www.rcsb.org/) was imported into the Pymol software to separate the receptor protein and the ligand molecule, followed by pretreatments such as dehydration. After importing the active components and corresponding potential therapeutic targets into Pyrx0.8 software for pretreatment and energy optimization, Autodock vina was used for molecular docking. The crystal box was selected to maximize the coverage of the entire receptor and active site. The binding affinities of receptors and ligands were analyzed and the docking results were compared with those of the original ligand.

### Preparation of *P. frutescens* Aqueous Extracts

*P. frutescens* leaves (lot no. 200501, place of origin: Liu’an, China) was purchased from Anhui Dabie Mountain Traditional Chinese Medicine Decoction Pieces Co. Ltd (Liu’an, China), and all herbs meet the standards of the Chinese Pharmacopoeia 2015. We weighed out 1200 × g of decoction pieces of *P. frutescens* leaves and extracted them twice with water. Firstly, the herb was immersed in 12,000 ml water and heated for 1 h at 100°C. 9000 ml of filtrate was obtained. After filtration, boiled herb was added 8000 ml water and heated for 1 h again. Then, we got 6800 ml of filtrate. The two filtrates were mixed and concentrated to obtain *P. frutescens* Aqueous Extracts (PFAE). Finally, PFAE solvent was prepared into freeze-dried powder by vacuum freeze-drying method. The PFAE freeze-dried powder was dissolved in water for use in the experiments. The quality and component validation were examined with fingerprint analysis *via* high performance liquid chromatograph (HPLC).

### HPLC Analysis

A Waters e2695 HPLC (on-line degasser, quaternary pump, automatic sampler, and UV detector), with a Kh-500de ultrasonic cleaner (Kunshan Hechuang Ultrasonic Instrument), Sartorius CPA225D electronic balance (Nanjing Yimanelli Instrument and Equipment), and a C18 (250 mm, 4.6 mm, 5 m) chromatographic column were used in this analysis. Luteolin (lot no. B20888, HPLC≥98%), rosmarinic acid (lot no. B20862, HPLC≥98%), caffeic acid (lot no. B50428, HPLC≥98%), perilla aldehyde (lot no. B21699, HPLC≥98%) and (+)-catechin (lot no. B21722, HPLC≥98%) were purchased from Shanghai Yuanye Biotechnology (Shanghai, China).

200 mg of PFAE freeze-dried powder was weighed and resolved in 1 ml 80% methanol, then eddy mixed for 10 min. After centrifugation at 13,000 rpm for 10 min, the supernatant was taken for HPLC analysis. Five reference sample substances were accurately weighed and dissolved in methanol to prepare a standard solution. The concentration of each component was 0.5 mg/ml.

The chromatographic method was as follows: A Dubhe C18 chromatographic column (4.6 mm × 250 mm, 5 μm) was employed. The mobile phase was acetonitrile (A) - 0.1% acetic acid (B). Linear gradient elution: 0–17 min, 15%A, 17–20 min, 15%A, 20–25 min, 20%A, 25–30 min, 20%A, 30–38 min, 28%A, 38–40 min, 28%A, 20%A, 40–45 min, 35% A, 45–50 min, 35%A ∼ 40%A, 50–60 min, 40%A ∼ 15%A. The volumetric flow rate was 1 ml/min, the column temperature was 25 C, the detection wavelength was 330 nm, and the operation time was 60 min. After each run, the chromatographic column was balanced with the initial mobile phase for 20 min to ensure the stability of peak extraction.

10 μl of the reference solution and test solution were accurately absorbed and injected into the liquid chromatograph. The chromatographic peaks, retention time and peak area were recorded within 60 min.

### Experimental Verification

We have screened and identified some components in *P. frutescens* through network pharmacology and HPLC. Therefore, we conducted *in vitro* experiments to further verify whether these components can effectively reduce kidney damage. Luteolin is a key component in network pharmacological model, and molecular docking model shows that it binds well to corresponding therapeutic targets. In addition, it has also been identified and quantified in the PFAE by HPLC. Therefore, luteolin was selected as the representative active component for subsequent *in vitro* experiments. According to the network pharmacological analysis, luteolin inhibits CKD progression mainly by regulating apoptosis. Previous studies have successfully established a tubular epithelial cell (TEC) injury model using adriamycin (ADR), which promotes TEC apoptosis. The model confirmed that the mechanism is closely related to oxidative stress and the mitogen-activated protein kinase (MAPK) pathway, which is an important form of renal pathology in CKD stage 3–5. Therefore, *in vitro*, ADR was used to disrupt renal TECs to construct a kidney injury model. Subsequent treatment with luteolin was performed to assess whether it can reduce apoptosis and regulate the expression of AKT, p53, and IL6. Oxidative stress, MAPK pathway and PI3K-AKT pathway are the most common and important ways related to apoptosis. Therefore, we further detected them to explore the mechanism by which luteolin reduces apoptosis.

### Reagents

Luteolin (lot no. HY-N0162, 98.15%) was purchased from MedChemExpress (Shanghai, China). ADR hydrochloride (lot no. S120812, 99.13%) was purchased from Selleck Chemicals (Shanghai, China). Fetal bovine serum (FBS) was purchased from Thermo Fisher Scientific (Waltham, MA, United States), as were phosphate buffered saline (PBS), 0.25% trypsin-EDTA, antibiotic-antimycotic (Anti-Anti, 100×), and Dulbecco’s modified Eagle medium/F-12 (DMEM/F-12). Cell Counting Kit-8 (CCK-8) was obtained from APExBIO (Houston, TX, United States). Dimethyl sulfoxide (DMSO) was purchased from Sigma-Aldrich (St. Louis, MO, United States). Antibodies to phosphorylated (p)-p38 MAPK (Thr180/Tyr182) (lot no. 4511), p-extracellular signal-regulated kinase (ERK)1/2 (Thr202/Tyr204) (lot no. 4370), p-stress-activated protein kinase/c-Jun N-terminal kinase (SAPK/JNK) (Thr183/Tyr185) (lot no. 4668), p-AKT (Ser473) (lot no. 4060), p53 (lot no. 2524), p-p53 (Ser15) (lot no. 9284), Caspase3 (lot no. 9662), and Cleaved Caspase-3 (lot no. 9664) were procured from Cell Signaling Technology (Shanghai, China). Antibodies to AKT (lot no. 10176-2-AP), p38 MAPK (lot no. 14064-1-AP), JNK (lot no. 24164-1-AP), Bcl-2 (lot no. 26593-1-AP), Bax (lot no. 50599-2-Ig), and β-actin (lot no. 66009-1-Ig), horseradish peroxidase (HRP)-conjugated Affinipure goat anti-mouse IgG (H+L) (lot no. SA00001-1), and HRP-conjugated Affinipure goat anti-rabbit IgG (H+L) (lot no. SA00001-2) were purchased from Proteintech (Wuhan, China). Antibodies to p-AKT (Thr308) (lot no. AF0016), p-phosphoinositide 3-kinase (PI3K) p85 (Tyr458) [Tyr467]/p55 (Tyr199) (lot no. AF0016), Interleukin 6 (IL6) (lot no. DF6087), ERK1/2 (lot no. AF0155) and poly (ADP-ribose) polymerase 1 (PARP1) (lot no. DF7198) were from Affinity Biosciences (Cincinnati, OH, United States). N-acetyl-L-cysteine (NAC) (lot no. S0077, >99%), reactive oxygen species (ROS) assay kit, BCA protein assay kit, One Step TdT-Mediated dUTP Nick End Labeling (TUNEL) Apoptosis Assay Kit, and 4,6-diamidino-2-phenylindole (DAPI) staining solution were purchased from Beyotime Biotechnology (Shanghai, China). The Annexin V fluorescein isothiocyanate/propidium iodide (FITC/PI) apoptosis kit was purchased from Multi Sciences (Hangzhou, China).

### Cell Line and Culture

Normal rat tubular proximal epithelial cell lines (NRK-52E) were obtained from the University of Yamanashi (Yamanashi, Japan). The cells were maintained in DMEM/F-12 supplemented with 5% FBS, 100 U/ml penicillin G, and 100 mg/ml streptomycin, and incubated at 37°C in 5% CO_2_. The cells at approximately 80–90% confluency were digested and passaged.

### Cell Morphology and Viability

NRK-52E cells were digested and seeded in 96-well plates at 1 × 10^4^ cells per well. After 24 h of incubation, the cells were cultured in a serum-free medium and stimulated with ADR (2 μg/ml) in the absence and presence of PFAE (5 mg/ml) or luteolin (10 μM/L). After 24 h of treatment, changes in cell morphology and number were observed by inverted phase-contrast microscopy using model CKX41 microscope (Olympus, Tokyo, Japan). Then, the medium was discarded, and 100 μl serum-free medium containing 10% CCK-8 was added to each well. After incubating for another 1 h, the absorbance was measured using a model ELx800 spectrometer (BioTek Instruments, Winooski, VT, United States) at a wavelength of 450 nm. Cell viability is expressed as a percentage of the control.

### Detection of Production of ROS

ROS production was detected using an ROS detection kit. Briefly, cells were seeded in a 12-well plate and incubated for 24 h. ADR, luteolin, and NAC were used to stimulate the cells according to the experimental protocol. The fluorescent probe 2ʹ,7ʹ-dichlorofluorescin diacetate (DCFH-DA) was diluted with serum-free medium at a ratio of 1:1000 to obtain a final concentration of 10 μmol/L. The medium was removed and 500 μl of diluted DCFH-DA was added to each well. Cells were incubated at 37°C for 20 min and washed thrice with serum-free medium. Fluorescent images were visualized and captured with a standard green fluorescence cube using a model Ts2R inverted fluorescence microscope (Nikon, Tokyo, Japan). Fluorescence images were analyzed quantitatively using ImageJ software (1.52a; NIH, Bethesda, MD, United States).

### Fluorescence Staining of Apoptotic Cells

TUNEL staining kit and DAPI staining solution were used to assess the presence of apoptotic cells. Briefly, the cells were incubated and treated in accordance with the experimental protocol, washed once with PBS, and fixed with 4% paraformaldehyde for 30 min. Then, they were permeabilized with 0.3% Triton X-100 in PBS for another 30 min and washed three times with PBS. Fifty microliters of TUNEL staining solution were added to each well and incubated in the dark for 1 h at 37°C. Then, cells were washed three times with PBS and 100 μl of DAPI staining solution was added to each well, followed by incubation in the dark for 10 min and washing with PBS. Finally, cell fluorescent images were captured using a fluorescence microscope (Nikon Ni-U, DS-Qi2) equipped with standard red and ultraviolet fluorescence cubes. Fluorescent images were analyzed quantitatively using ImageJ software (1.52a).

### Flow Cytometry Analysis to Detect Apoptosis

An Annexin V-FITC/PI apoptosis assay kit was used to detect apoptosis. Briefly, the cells were washed with PBS and digested with trypsin for 3 min. Then, serum-containing medium was added to terminate the digestion. Each sample was centrifuged and the supernatant was discarded. Five hundred microliters of 1× binding buffer were added to resuspend the cells, followed by 5 μl Annexin V-FITC and 10 μl PI to each tube. After incubation for 10 min protected from light on ice, a FACSCelesta flow cytometer (BD, Santa Clara, CA, United States) was used to analyze the percentage of apoptotic cells.

### Western Blot Analysis

After incubation and intervention, the cells were lysed in RIPA buffer supplemented with 2% protease and phosphatase inhibitor cocktail for 20 min on ice. After sonication, the samples were centrifuged at 4°C for 20 min, and each supernatant was collected for subsequent experiments. The total protein concentration was determined using a BCA protein assay kit. Equal amounts of protein (20 µg) were separated by 10 or 12% sodium-dodecyl sulfate-polyacrylamide gel electrophoresis (SDS-PAGE). The resolved proteins were electrophoretically transferred to polyvinylidene difluoride (PVDF) membranes (Millipore, Billerica, MA, United States). The membranes were blocked with 5% non-fat dried milk in PBS containing Tween (PBST) buffer for 1 h at room temperature, washed thrice with PBST, and incubated with primary antibodies overnight at 4°C. The membranes were washed again and incubated with HRP-conjugated anti-rabbit or anti-mouse IgG for 1 h at room temperature. The bands were flushed with chemiluminescent HRP substrate and imaged using a chemiluminescence system (ChemiDoc™ XRS+ with Image Lab Software; Bio-Rad, Hercules, CA, United States). Western blot images were analyzed quantitatively using ImageJ software (1.52a). β-actin was also assayed to confirm equal loading of proteins.

### Statistical Analyses

Statistical analyses were performed using SPSS version 19.0 (IBM, Armonk, NY, United States). All values are expressed as mean ± standard deviation (SD). Comparisons of two populations were made using unpaired 2-tailed Student’s t-test. For multiple groups, statistical significance was determined using one-way analysis of variance (ANOVA) followed by Dunnett’s test. Statistical significance was set at *p*<0.05.

## Results

### Main Active Components and Targets of *P. frutescens*

A total of 328 chemical components of *P. frutescens* were retrieved from the TCMSP database. Use of OB ≥30% and DL ≥0.18 as the screening criteria yielded 14 active components. An analysis of the published literature revealed that although (s) – carvone (Alasanea and Liu, 2017), caffeic acid (Tyszka-Czochara et al., 2018), rosmarinic acid (Colica et al., 2018), caryophellene (Sharma et al., 2016), perilla aldehyde (Song et al., 2018), and perilla ketone (Roellecke et al., 2017) did not meet the aforementioned OB and DL screening criteria, *in vivo* and *in vitro* experiments were validated. Therefore, these components were included to result in 19 main active components (C20 Supraene did not retrieve the predicted target, and so was excluded in further research) (Table 1 and Supplementary Figure S1). The predicted targets of the active components were searched for in the TCMSP database, and the predicted targets in the Uniport database were checked to obtain 225 targets of the main active components. Of these, 141 unique targets were finally obtained after deduplication.

**TABLE 1:** Main active components of *P. frutescens*.

Code	Compound name	CAS	OB%	DL
C1	Bishomo-gamma-linolenic acid	1783-84-2	44.11	0.2
C2	(s)-Carvone	2244-16-8	47.43	0.03
C3	11,14-Eicosadienoic acid, methyl ester	2463-02-7	39.67	0.23
C4	ZINC03860434	117-81-7	43.59	0.35
C5	Beta-sitosterol	83-46-5	36.91	0.75
C6	Beta-carotene	7235-40-7	37.18	0.58
C7	Clionasterol	83-47-6	36.91	0.75
C8	Cholesterin	57-88-5	37.87	0.68
C9	Cirtrusin C	18604-50-7	40.52	0.23
C10	(+)-Catechin	154-23-4	54.83	0.24
C11	Cyanin	523-42-2	47.42	0.76
C12	Gondoic acid	5561-99-9	30.7	0.2
C13	Caffeic acid	501-16-6	25.76	0.05
C14	Rosmarinic acid	20283-92-5	1.38	0.35
C15	Luteolin	491-70-3	36.16	0.25
C16	Caryophellene	87-44-5	23.79	0.09
C17	Linolenic acid ethyl ester	1191-41-9	46.1	0.2
C18	Perilla aldehyde	18031-40-8	39	0.03
C19	Perilla ketone	553-84-4	81.94	0.03

OB, oral bioavailability; DL, drug-likeness.

### Main Targets Related to CKD

The relevant targets of CKD stage 3–5/CRF were searched for in the Gene cards database with a score ≥10 as the screening criterion. The search resulted in 2263 main targets.

### Key Targets of *P. frutescens* in Treatment of CKD

**FIGURE 1:**
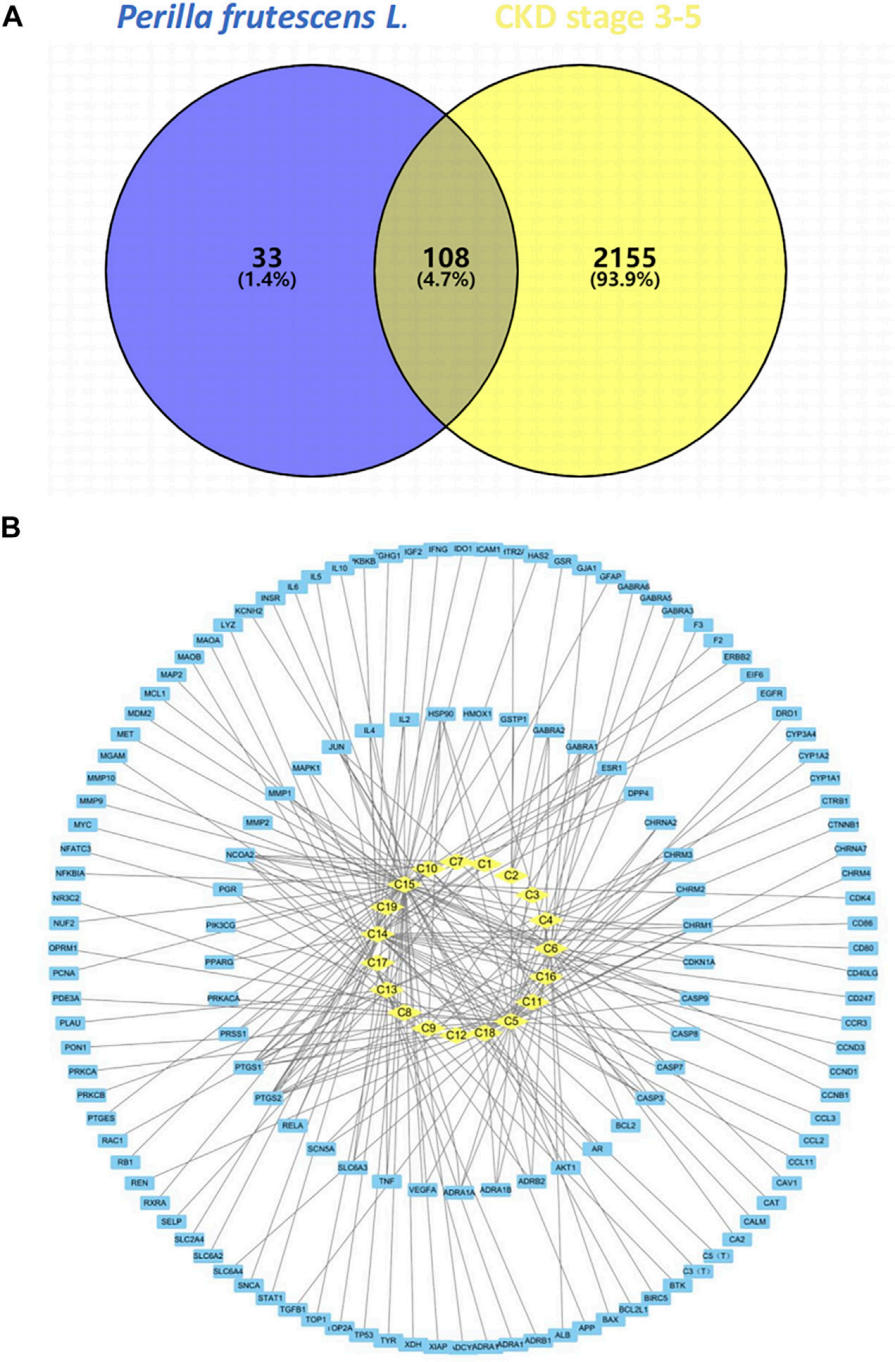
Construction and analysis of the network model of Perilla frutescens *L*. in the treatment of chronic kidney disease (CKD). **(A)** Relevant targets of *P. frutescens* in the treatment of CKD stages 3–5. The predicted targets of main active components were mapped to the main targets of CKD stages 3–5, and 108 relevant targets were obtained. **(B)** Main active components-targets network model. The main active components, targets, and their interaction relationships were imported into Cytoscape3.6.0 software to construct the network model. The yellow diamond represents the active components, the blue square represents the targets, and the lines represents the interaction relationships.

The predicted targets of *P. frutescens* were mapped to the main targets related to CKD stage 3–5 using Venny2.1.0 software. A Venn diagram was constructed (Figure 1A). A total of 108 intersection targets were obtained. These may be the key targets of *P. frutescens* for CKD treatment.

### Main Active Components-Targets Network Model

The main active components and key targets of *P. frutescens* were imported into Cytoscape 3.6.0, to build a main active component-target network model. The model included 160 nodes and 225 edges. In this model, 19 active components are shown as diamond-shaped yellow nodes, 141 targets are blue square nodes, and the edges represent the interaction between the components and the targets (Figure 1B). The Network Analyzer plugin was used to perform network topology analysis and to calculate the degree, betweenness centrality and closeness centrality parameter values. The median of the degree was one. Therefore, we chose degree >2 (i.e., more than twice the median) as the criterion to filter out the core nodes in the network model, including 12 components and 18 targets (Supplementary Table S1).

Further analysis of the nodes in the table revealed that the highest degree among the main active components was C15 (luteolin), with 57 targets. In descending order, C5 (β-sitosterol) corresponded to 37 targets, C14 (rosmarinic acid) had 32 targets, C13 (caffeic acid) corresponded to 22 targets, and C6 (β-carotene) had 21 targets. The target is the key to the pharmacological effect of the component, so it is speculated that luteolin, β-sitosterol, and rosmarinic acid are the key components of *P. frutescens* in the treatment of CKD stage 3–5. Among the target nodes, the highest degree was prostaglandin-endoperoxide synthase 2 (PTGS2), which targeted 11 components. Subsequently, in descending order, PTGS1 and nuclear receptor coactivator 2 (NCOA2) all targeted eight components, heat shock protein 90 (HSP90) is connected to five active components. The biological functions related to these key targets were mainly related to inflammation, immunity, metabolism, and others, suggesting that *P. frutescens* may exert its pharmacological effects on CKD through the aforementioned approaches.

Additionally, the network model found that 18 active components targeted at least two targets, and 41 targets were connected to at least two components. There was a large amount of cross-coincidence between each component and target. These results reflect the multi-component, multi-target effects of *P. frutescens*.

### PPI Network Model

**FIGURE 2:**
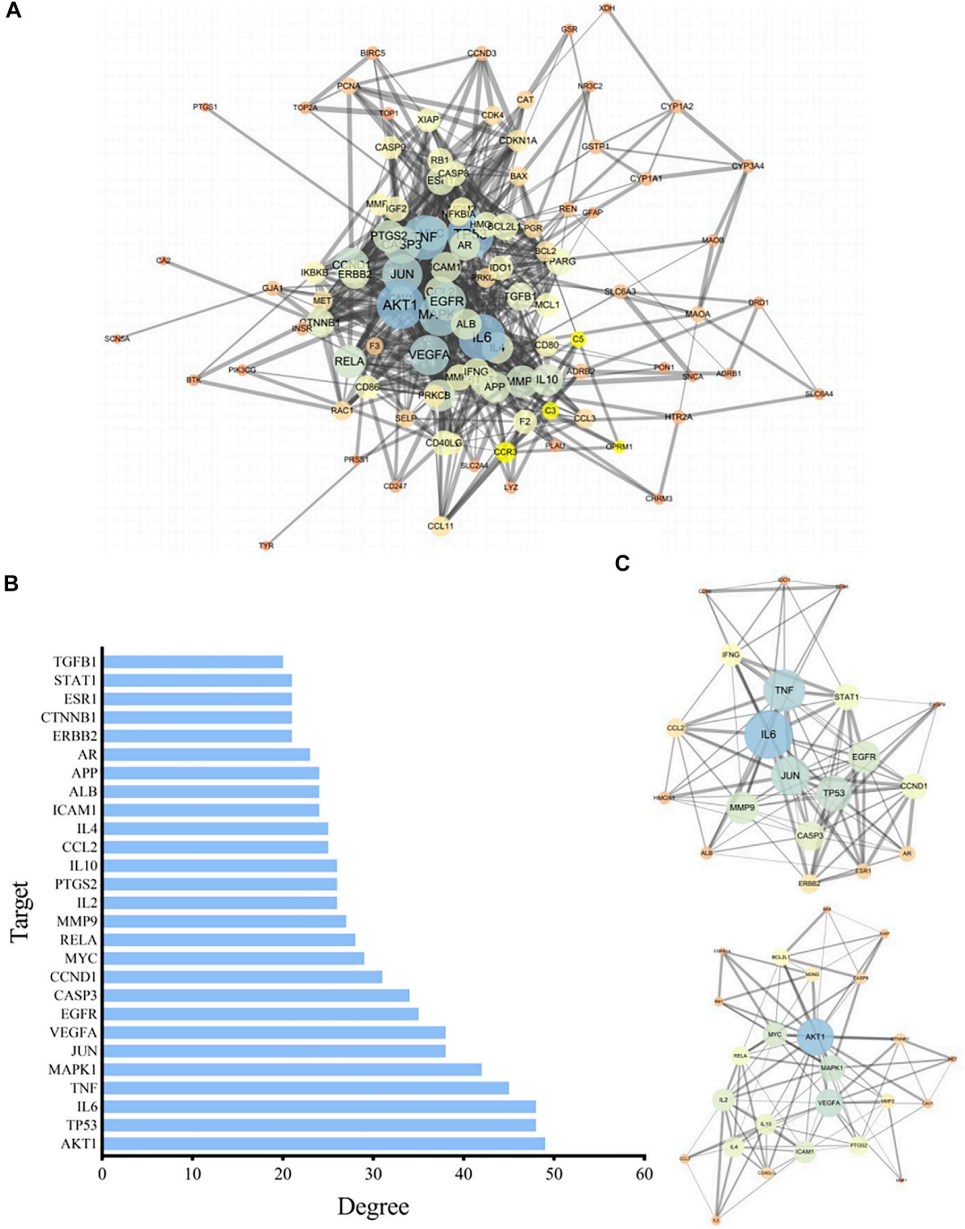
Construction and analysis of the protein-protein interaction (PPI) network. **(A)** The PPI network model. The related targets of *P. frutescens* for the treatment of CKD stages 3–5 were imported into the String 11.0 database to construct the PPI network. The PPI network information was input into Cytoscape 3.6.0 software to construct the PPI network model and adjusted according to the network topology analysis. **(B)** Key nodes in PPI network model. The key nodes in the PPI network model were screened with Degree ≥20, 27 key proteins in the PPI network were selected. **(C)** Key modules in the PPI network model. Modular analysis of the PPI network was carried out by using MCODE plugin. Two key protein modules were screened. Module A contains 20 targets and 101 interactions and module B contains 25 targets and 99 interactions.

The relevant targets of *P. frutescens* for treating CKD stage 3–5 were imported into String 11.0, under the condition of “homo sapiens.” The highest confidence protein score value was set as >0.7, a PPI network was constructed, and unrelated individual proteins were removed. The network included 105 targets and 736 interactions. The PPI network information was imported into Cytoscape3.6.0 to build a network model, and the Network Analyzer plugin was used to analyze network characteristics. By adjusting the node color and size according to the degree, the greater the degree, the larger was the node. The thickness of the side was revised according to the Combine score: the larger the Combine score, the thicker the side (Figure 2A).

We selected degree ≥20 as the criterion. Twenty-seven key proteins in the PPI network were selected (Figure 2B), including AKT1, tumor protein 53 (TP53), IL6, tumor necrosis factor (TNF), MAPK1, JUN, vascular endothelial growth factor A (VEGFA), epidermal growth factor receptor (EGFR), caspase 3 (CASP3), cyclin D1 (CCND1), MYC, RELA, matrix metalloproteinase 9 (MMP9), IL2, PTGS2, and others, implicating these targets as the main targets of *P. frutescens* in the treatment of CKD stage 3–5.

The PPI network is modular, and the functional module is the basic unit of protein function. Modular analysis can simplify the complex protein network, which is conducive to further research on the biological and pharmacological functions of the protein. Therefore, the MCODE plugin in the Cytoscape software was used to perform modular analysis of the PPI network, with Degree Cutoff = 2, Node Score Cutoff = 0.2, K-Core = 5, and Max. Depth = 100. Two key protein modules were identified. Protein module A contained 20 targets and 101 interactions. Protein module B contained 25 targets and 99 interactions (Figure 2C).

### Enriched Gene Biological Functions and Signaling Pathways

**FIGURE 3:**
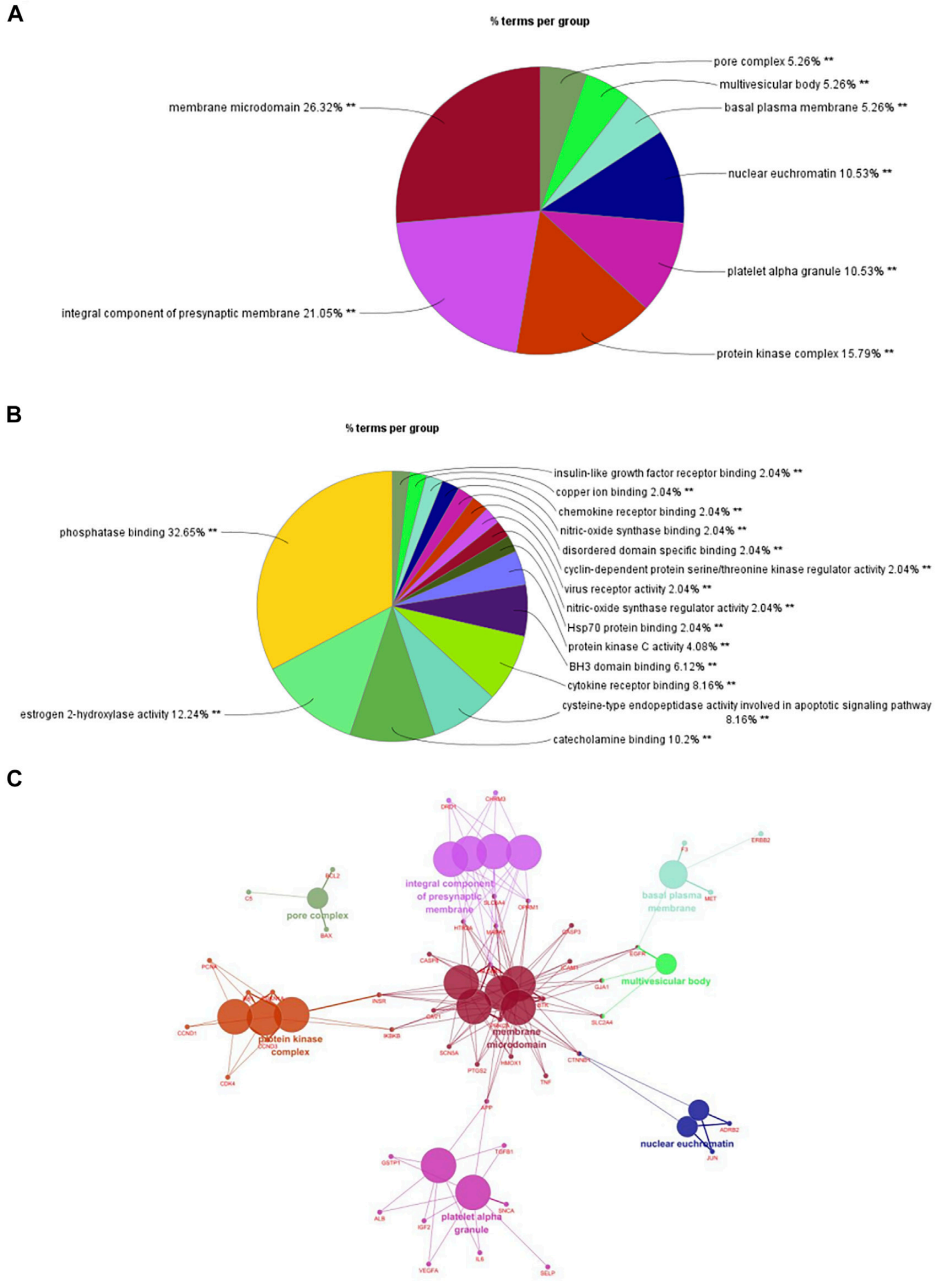
Enrichment analysis of cellular component (CC) and molecular function (MF). **(A)** Enrichment analysis of CC. Targets (*n* = −105) in the PPI network were imported into Cytoscape 3.6.0 software. The enrichment analysis of CC was carried out with a *p*-value ≤0.01. A pie chart and network model of the mainly enriched related CCs were obtained. **(B)** Enrichment analysis of MF. According to the same methods and criteria of CC enrichment analysis, the enrichment analysis of MF was carried out to obtain the main related MFs. **(C)** The network model of CC enrichment analysis. The enrichment analysis of CC was selected as a representative to construct the network model by using Cytoscape 3.6.0 software.

We imported 105 targets in the PPI network into Cytoscape3.6.0 software and used ClueGO and CluePedia plugins for enrichment analysis of CC and MF. A *p* ≤ 0.01 was the screening parameter. A pie chart was drawn (Figures 3A,B). CCs mainly included membrane microdomains, integral components of presynaptic membranes, protein kinase complexes, platelet alpha granules, nuclear euchromatin, basal plasma membrane, multivesicular body, and pore complex. MFs mainly involved phosphatase binding, estrogen 2-hydroxylase activity, catecholamine binding, cysteine-type endopeptidase activity involved in the apoptotic signaling pathway, cytokine receptor binding, BH3 domain binding, protein kinase C activity, HSP70 protein binding, nitric oxide synthase regulator activity, virus receptor activity, cyclin-dependent protein serine/threonine kinase regulator activity, disordered domain-specific binding, nitric oxide synthase binding, chemokine receptor binding, copper ion binding, and insulin-like growth factor receptor binding. Enrichment analysis of CCs was selected as a representative to construct a network model (Figure 3C). Multiple targets were connected to at least two CCs, which are the key nodes in the network. The findings provided further evidence of the multi-target, multi-effect characteristics of *P. frutescens*.

**FIGURE 4:**
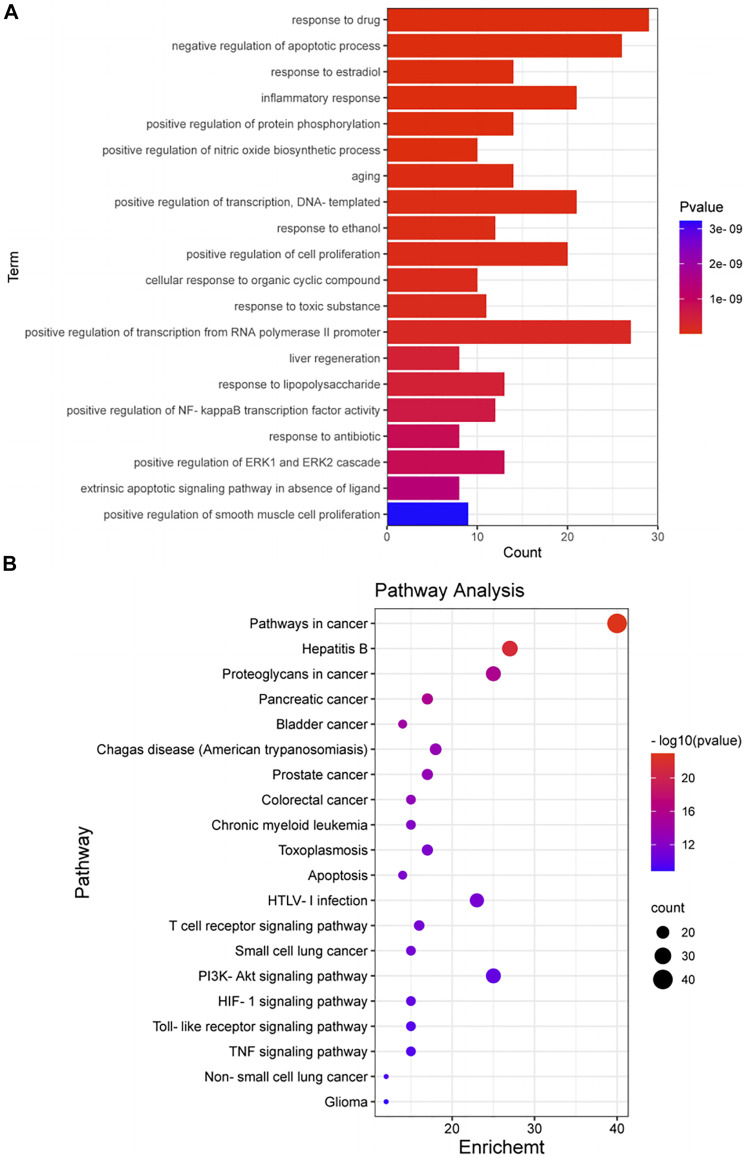
Enrichment analysis of biological process (BP) and KEGG signaling pathway. **(A)** Enrichment analysis of BP. The 105 targets in PPI network were imported into David6.8 online analysis platform for enrichment analysis of BP. The top 20 BPs were selected to draw the bar graph. **(B)** Enrichment analysis of KEGG signaling pathway. The analysis was carried out according to the same methods and criteria of BP enrichment analysis. The top 20 signaling pathways were selected to draw the bubble diagram.

A total of 105 targets were imported into the DAVID6.8 online analysis platform for the enrichment analysis of BP and KEGG signaling pathways. With *p* < 0.01 as the screening criterion, 254 BPs and 101 pathways were obtained. The first 20 BPs and pathways were selected using the clusterProfiler package in R language to draw bar graphs and bubble diagrams (Figures 4A,B and Supplementary Figures S2A,B). In the bubble diagram, the node size represents the gene number: the larger the node, the more genes corresponding to the node. The color of the node represents the *p*-value. The smaller the *p*-value, the redder the color; otherwise, the values were depicted in shades of blue. The ordinate represents the BP or pathway name corresponding to the node, and the abscissa represents the fold enrichment. The greater the fold enrichment, the higher the degree of gene enrichment. BPs were observed to be mainly related to the response to drugs, negative regulation of apoptotic process, response to estradiol, inflammatory response, positive regulation of protein phosphorylation, positive regulation of nitric oxide biosynthesis process, aging, positive regulation of transcription, response to ethanol, positive regulation of cell proliferation, cellular response to organic cyclic compounds, response to toxic substances, positive regulation of transcription from RNA polymerase II promoter, positive regulation of nuclear factor-kappa B (NF-κB) transcription factor activity, and positive regulation of ERK1 and ERK2 cascade. Enriched pathways related to CKD stages 3–5 mainly included the apoptosis signaling pathway, T cell receptor (TCR) signaling pathway, PI3K-AKT signaling pathway, hypoxia inducible factor-1 (HIF-1) signaling pathway, Toll-like receptor (TLR) signaling pathway, and TNF signaling pathway. Thus, *P. frutescens* may play a therapeutic role in CKD stages 3–5 by regulating the above multiple pathways.

### Docking of Core Components and Corresponding Main Targets of *P. frutescens*

**FIGURE 5:**
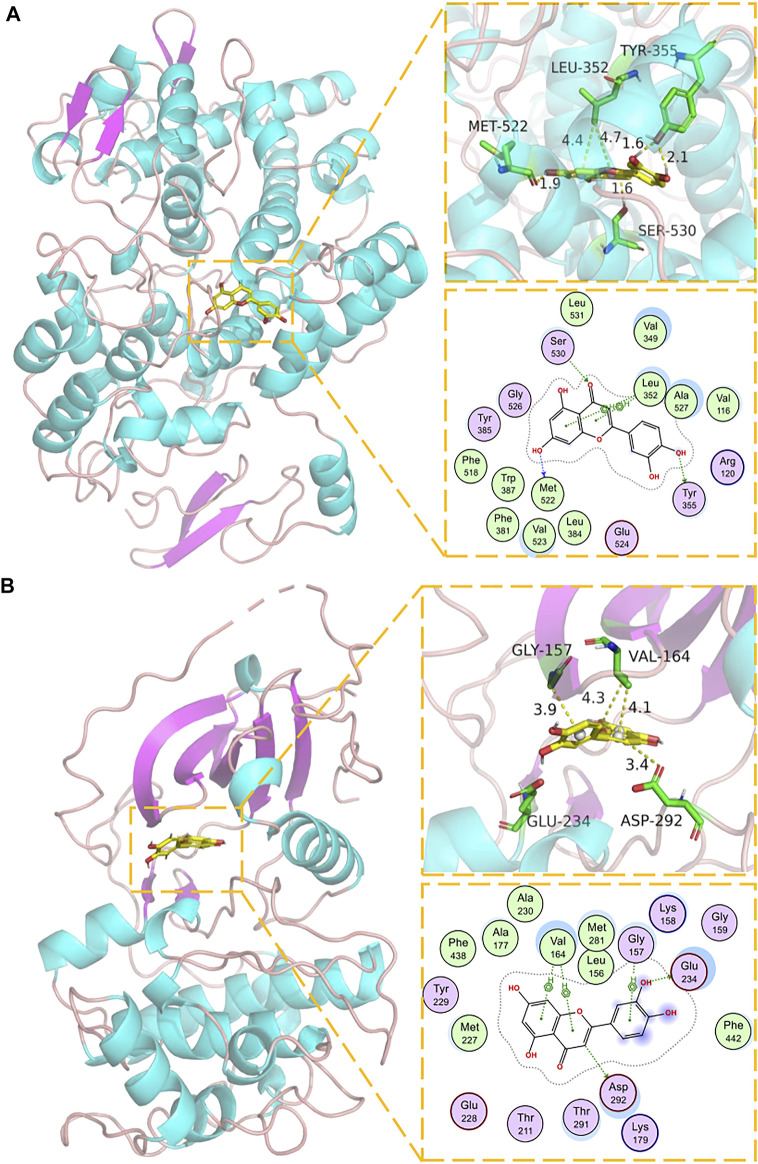
Molecular docking model of luteolin and corresponding targets. **(A)** Molecular docking mode of luteolin and PTGS1. The backbone of protein was rendered in tube and colored in bright blue. Luteolin is rendering by yellow. Yellow dash represents hydrogen bond distance or π-stacking distance. **(B)** Molecular docking mode of luteolin and AKTI. The backbone of protein was rendered in tube and colored in bright blue. Luteolin is rendering by yellow. Yellow dash represents hydrogen bond distance or π-stacking distance.

The key components in the component-target network were selected as the ligand, and the corresponding core node as the receptor. Imported Pyrx was used for preprocessing, and Autodock vina was used for molecular docking (Table 2). Binding affinity <0 meant that the receptor and ligand bound spontaneously: the lower the value, the greater the binding affinity. The binding energies of the targets, PTGS2, AKT1, p53, TNF-α, MAPK1, IL6, JUN, and their original ligands were −11.6, −8.7, −4.3, −9.7, −9.8, −4.5, −9.6 kJ/mol, respectively. The binding capacity of each key component and corresponding target was similar to that of the original ligand. We selected luteolin, the most critical component in the network model, and performed molecular docking and visual analysis through Schrödinger Maestro and Pymol2.1 (Figures 5A,B). These results verified that the main active components of *P. frutescens* may play a therapeutic role in CKD by acting on their corresponding key targets.

**TABLE 2:** Molecular docking of active components and corresponding targets in *P. frutescens*.

Component	Target	Binding Affinity (kJ/mol)
Luteolin	PTGS2	−9.3
	AKT1	−8.5
	p53	−6.9
	TNF-α	−8.9
	MAPK1	−8.8
	IL6	−7.5
	JUN	−8.7
β-sitosterol	PTGS2	−11.8
	JUN	−11.6
Rosmarinic acid	PTGS2	−6.7
	MAPK1	−7.9
Caffeic acid	PTGS2	−7.2
	TNF-α	−6.8
β-carotene	PTGS2	−9.0
	AKT1	−8.9
	JUN	−9.5

### Quality Control and Component Validation of PFAE

**FIGURE 6:**
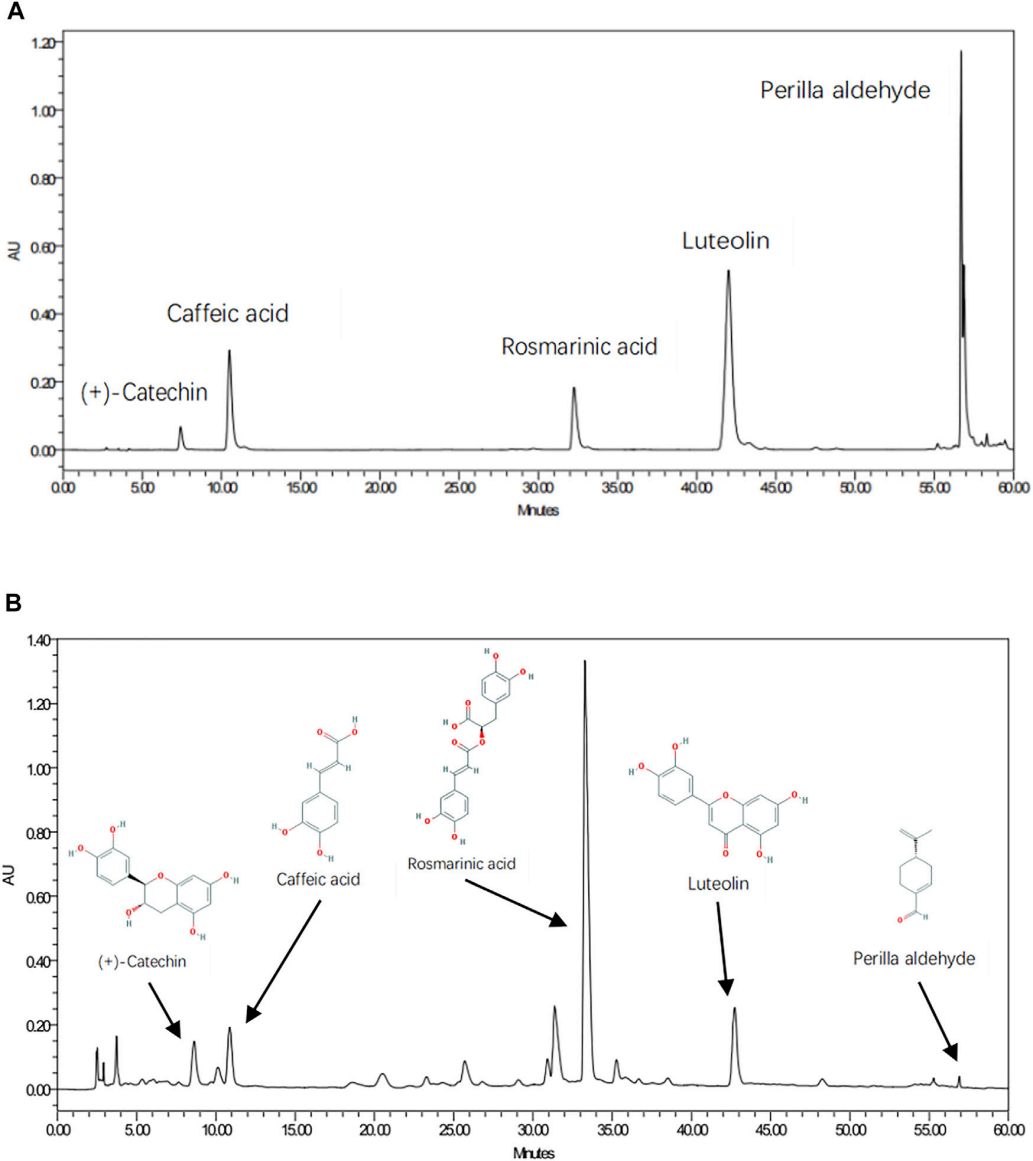
Quality Control and Component Validation of P. frutescens Aqueous Extracts (PFAE) by HPLC. **(A)** Chromatograms of mixed standards. **(B)** PFAE samples.

As shown in Figure 6, five chemical components in PFAE were detected by HPLC, including luteolin (C_15_H_10_O_6_; CAS: 491-70-3), rosmarinic acid (C_18_H_16_O_8_; CAS: 20283-92-5), caffeic acid (C_9_H_8_O_4_; CAS: 501-16-6), perilla aldehyde (C_10_H_14_O; CAS: 18031-40-8) and (+)-catechin (C_15_H_14_O_6_; CAS: 154-23-4). Luteolin, rosmarinic acid and caffeic acid are the main active components of *P. frutescens*, and the content is large. Therefore, the concentrations of these three components in different preparations were measured for quality control.

In addition, these components identified by HPLC were coincident with those screened from the TCMSP database. And according to the results of network pharmacological analysis, these five components were also the main active components in the network model of *P. frutescens* for treating CKD.

### Luteolin Reduces Adriamycin-Induced Renal Tubular Cell Injury

**FIGURE 7:**
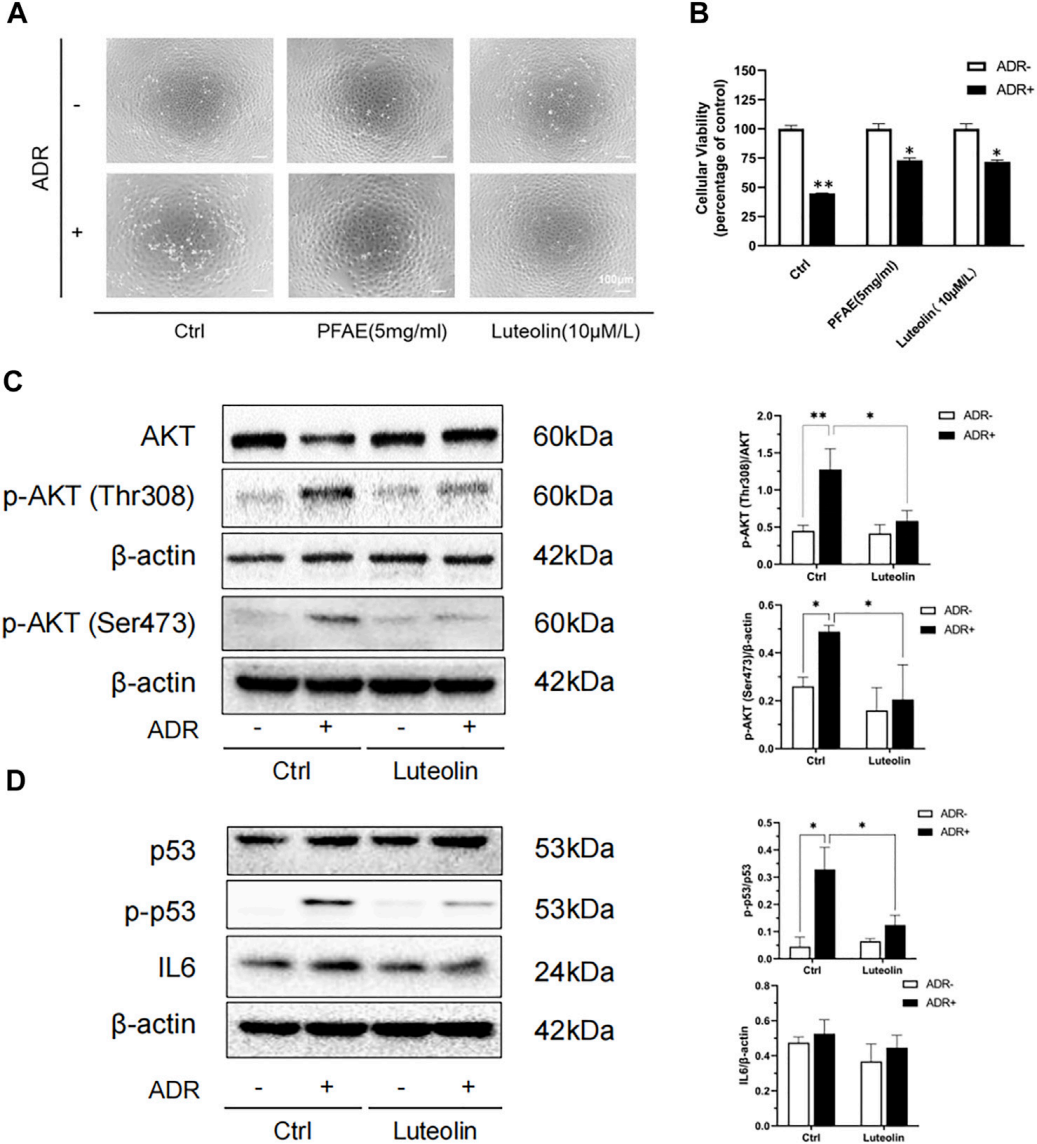
Luteolin alleviates Adriamycin (ADR)-induced renal injury and modulates AKT phosphorylation. **(A)** Effects of luteolin on the morphological changes of ADR-induced NRK-52E cells. Pretreated NRK-52E cells with FPAE (5 mg/ml) or luteolin (10 μM/L) for 1 h and then stimulated with ADR (2 μg/ml) for 24 h. Morphological changes of cells were observed by inverted phase-contrast microscopy (magnification ×100). **(B)** Effects of luteolin on cellular viability of ADR injury model. NRK-52E cells were exposed to the indicated concentrations of ADR with or without luteolin Cell viability was measured by the CCK-8 assay. Data are expressed as the percentages of living cells against the control (mean ± SD, *n* = 3; *p <0.05 versus ADR in Ctrl. **p <0.01 versus Ctrl) **(C)** Effects of luteolin on the phosphorylation of AKT in ADR-injured cells. NRK-52E cells were pretreated with luteolin for 1 h and then challenged with ADR for another 24 h. Cellular lysates were subjected to western blot analysis of AKT, phosphorylated AKT and B actin Statistical analysis of phosphorylated AKT is shown on the right (mean ± SD, *n* = 3; *p <0.05, **p <0.01) **(D)** Effects of luteolin on the expression of p53 and IL6 in ADR-injured cells. NRK-52E cells were pretreated with luteolin for 1 h and then incubated with ADR for another 6 h. Cellular lysates were subjected to western blot analysis of p53, phosphorylated p53, IL6 and β-actin. Statistical analysis of phosphorylated p53 and IL6 are shown on the right (mean ± SD, *n* = 3; *p <0.05).

According to the HPLC results, we calculated that the concentration of luteolin in the PFAE was about 0.15 mg/ml, which provided the basis for further study of its therapeutic effects. Luteolin-pretreated NRK-52E cells were examined to assess the effect on cell morphology and viability. Compared with the control group, the number of cells in the ADR (2 μg/ml) group was reduced, the cells were shrunken and deformed, and some damaged cells floated in the culture (Supplementary Figures S3A,B). Compared with the ADR group, the number of cells was increased in the PFAE (5 mg/ml) and luteolin (10 μM/L) pretreatment groups, and the cell morphology was normal (Figure 7A). Cell viability was measured using the CCK-8 assay. Compared with the ADR group, the cell viability in the PFAE+ADR and luteolin+ADR groups was significantly increased. Therefore, PFAE and luteolin (the key component of *P. frutescens*) can attenuate ADR-induced cell injury (Figure 7B).

### Luteolin Modulates the Phosphorylation of AKT and P53

AKT participates in a variety of physiological processes in the body, including cell proliferation, apoptosis, protein translation, and glucose metabolism. It plays an important role in various kidney diseases. AKT is the core protein in the PPI network, and molecular docking has revealed good binding ability between luteolin and AKT. We used ADR to construct an *in vitro* renal tubular injury model and found that it can significantly increase the phosphorylation of AKT. Upon the addition of luteolin was added, and this promotion was effectively inhibited (Figure 7C).

At the same time, TP53 and IL6 are also key targets of the PPI network model. P53 (the expressed protein of TP53) is a key protein that regulates the cell cycle. It is closely related to cell self-repair and cell apoptosis. IL6 is regarded as a pro-inflammatory cytokine, which plays a key role in the immune response and inflammation. Both p53 and IL6 are involved in the development of CKD, and can aggravate renal injury by inducing apoptosis, promoting inflammation, accelerating cell senescence and other ways (Amdur et al., 2016; Juvet et al., 2020; Wang et al., 2020). Therefore, we also detected the phosphorylation level of p53 and the expression of IL6. The results showed that luteolin can significantly reduce ADR-induced p53 phosphorylation, but had no significant effect on IL6 (Figure 7D). These results indicate that AKT and p53 may be the targets of luteolin in reducing ADR-mediated kidney damage.

### Luteolin Mitigates ADR-Elicited Apoptosis

**FIGURE 8:**
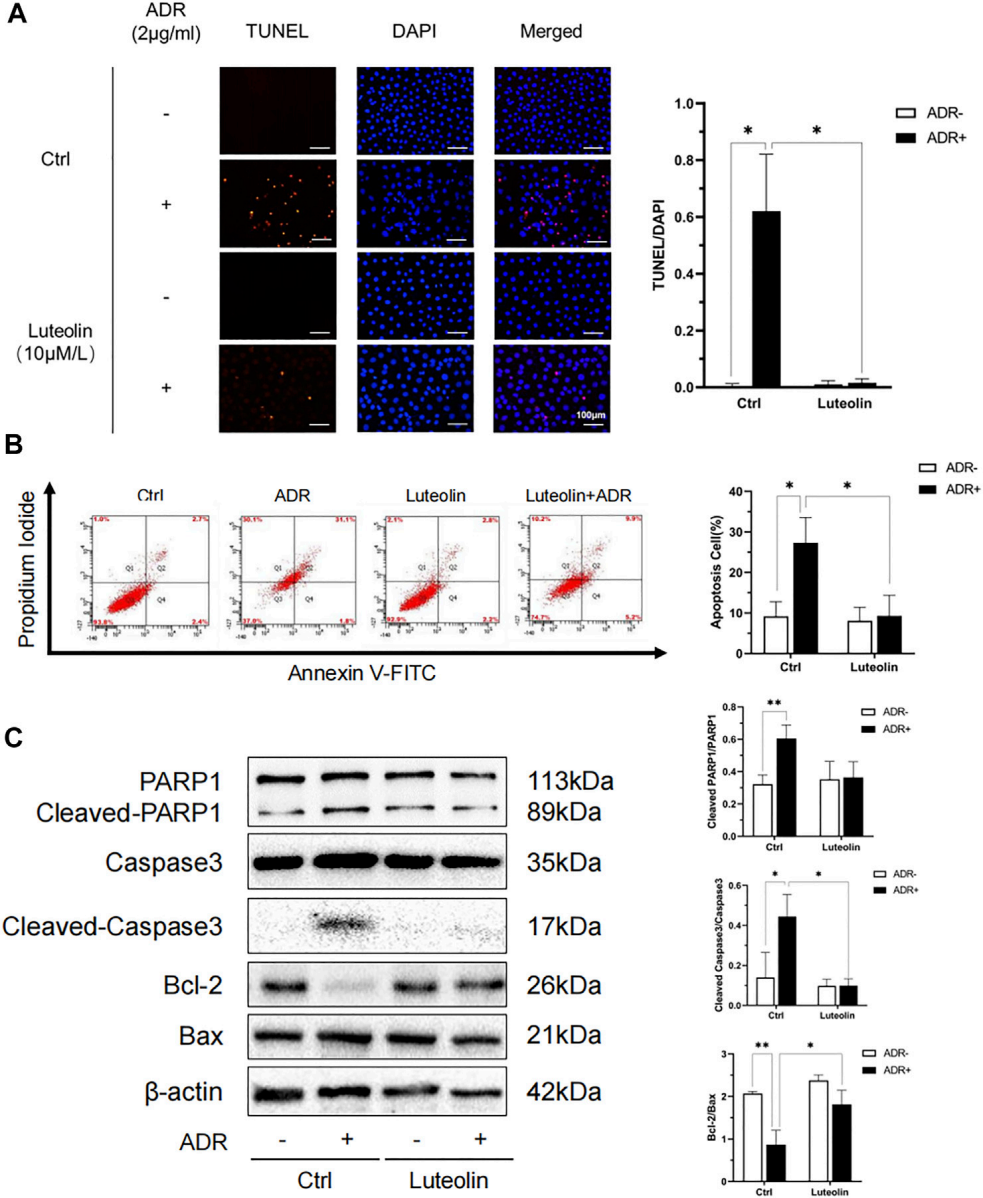
Luteolin reduces ADR-induced apoptosis. **(A)** Effects of luteolin in ADR-induced apoptosis by TUNEL and DAPI staining assay. NRK-52E cells were incubated with ADR for 24 h in the presence and absence of luteolin. Apoptotic cells were detected by TUNEL and DAPI staining. Fluorescence staining was observed by fluorescence microscope (magnification ×200) and the analysis result on the right was expressed as the percentages of dead cells compared with the Ctrl (mean ± SD, *n* = 3; *p <0.05) **(B)** Effects of luteolin in apoptosis as measured by flow cytometry. NRK-52E cells were pretreated with luteolin for 1 h and stimulated with ADR for another 24 h. Apoptotic cells were detected by flow cytometry using Annexin V-FITC/PI. Flow cytometry data is shown on the right (mean ± SD, *n* = 3; *p <0.05). **(C)** Effects of luteolin on expression of apoptosis-related proteins. NRK-52E cells were pretreated with luteolin for 1 h and incubated with ADR for another 24 h. Cellular lysates were analyzed by western blot targeting Cleaved-PARPI, Cleaved Caspase3, Bcl-2 and Bax. Statistical analysis of western blot results are shown on the right (mean ± SD, *n* = 3; *p* <0.05, **p <0.01).

TUNEL + DAPI staining and flow cytometry analysis were performed to verify the anti-apoptotic role of luteolin in NRK-52E cells treated with ADR. The apoptosis rate in the luteolin+ADR group was significantly lower than that in the ADR group (Figures 8A,B). We further tested the expression of apoptosis-related proteins, including PARP1, Caspase3, Bcl-2, and Bax. Compared with the ADR group, these apoptotic proteins were significantly down-regulated in the luteolin+ADR group (Figure 8C). These data show that luteolin mitigated ADR-induced apoptosis.

### Luteolin Relieves Oxidative Stress Induced by ADR

**FIGURE 9:**
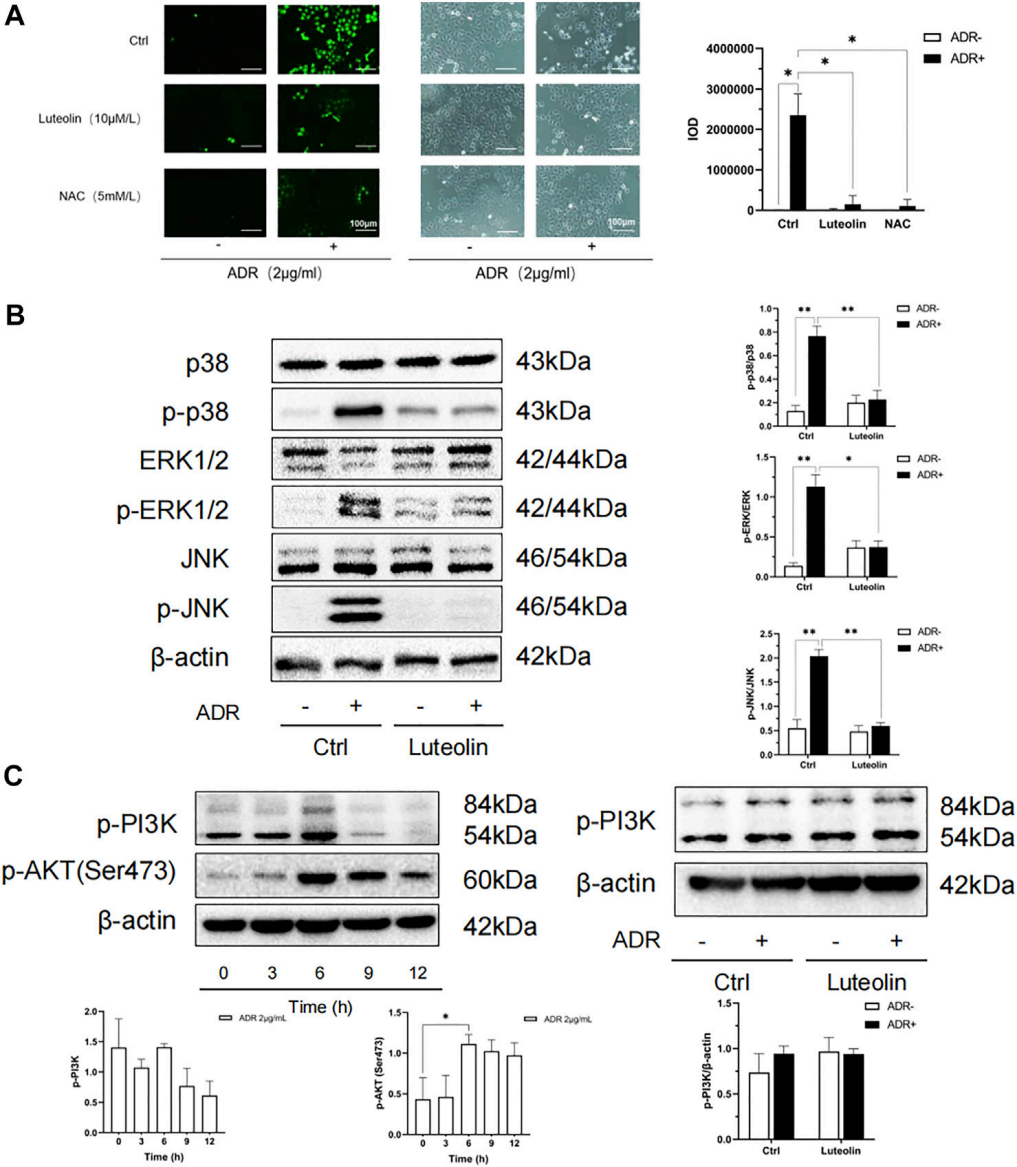
Oxidative stress and MAPK pathway may be the mechanism of luteolin to ameliorate apoptosis. **(A)** Effects of luteolin on ROS production. NRK-52E cells were pretreated with luteolin and NAC (5 mM/L) for 1 h and then stimulated with ADR for another 4 h. ROS staining was observed by florescence microscope (magnification ×200). The fluorescence intensity represents the ROS production level. Statistical analysis data is shown at the bottom (mean ± SD, *n* = 3; *p <0.05). **(B)** The regulatory effects of luteolin on MAPK pathway related proteins. NRK-52E cells were pretreated with luteolin for 1 h and incubated with ADR for 24 h. Cellular lysates were subjected to western blot analysis for phosphorylated p38 ERK1/2 and JNK. Densitometric analysis of these phosphorylated proteins are shown on the right (mean ± SD, *n* = 3; *p <0.05, **p <0.01). **(C)** Effects of ADR on phosphorylated PI3K and AKT. NRK-52E cells were stimulated with ADR for different periods. Then, the expression of phosphorylated PI3K and AKT was detected by western blot Densitometric analysis of these proteins are shown at the bottom (mean ± SD, *n* = 3; *p <0.05). **(D)** The regulatory effects of luteolin on p-PI3K. NRK-52E cells were pretreated with luteolin for 1 h and stimulated with ADR for another 6 h Cellular lysates were subjected to western blot analysis for phosphorylated PI3K. Densitometric analysis is shown at the bottom (mean ± SD, *n* = 3).

Oxidative stress is one of the important mechanisms in cell apoptosis and plays a key role in the pathological process of CKD. The overproduction of ROS is the initial factor of oxidative stress. We tested ROS production using an immunofluorescence probe. As shown in Figure 9A, after 4 h of incubation, ADR significantly increased the generation of ROS (green), whereas pretreatment with luteolin or NAC antioxidant suppressed the ADR-induced overproduction of ROS. Furthermore, compared with the NAC pretreated group, there was no significant difference in the inhibitory effect of luteolin on ROS induced by ADR. These outcomes suggest that luteolin effectively inhibits ADR-induced apoptosis *via* suppression of ROS overproduction.

### Luteolin Attenuated ADR-Induced Apoptosis by Regulating MAPK and P53 Pathways

According to the results of network pharmacology analysis, luteolin inhibits apoptosis to protect the kidneys against multiple insults. The MAPKs are critical in apoptosis and usually activated by oxidative stress. Thus, we focused on the MAPK pathway to assess the role of luteolin. As shown in Figure 9B, ADR significantly promoted the activation of MAPK families (ERK1/2, p38, and JNK) in renal tubular cells, whereas pretreatment of cells with luteolin effectively reduced the phosphorylation of p38, ERK1/2, and JNK. These results suggest that the regulation of MAPK pathways may mediate the protective effect of luteolin on ADR-induced kidney damage, and can be regarded as a link between oxidative stress and apoptosis.

PI3K-Akt pathway plays an important role in cell apoptosis, and it is also one of the top pathways in the KEGG enrichment analysis. We have found that luteolin can reduce the phosphorylation of AKT. Therefore, we further detected the phosphorylation of PI3K. The results showed that the effect of luteolin on PI3K phosphorylation did not reach statistical significance (Figures 9C,D). According to the results, the role of PI3K-Akt pathway in luteolin’s reduction of apoptosis has not been clarified, and the reason why the changes of PI3K and AKT are not completely parallel needs to be further studied.

The p53 pathway is also closely related to cell apoptosis. In Figure 7D, we have found that luteolin can inhibit the phosphorylation of P53. Therefore, down-regulation of the p53 pathway may also be an important mechanism by which luteolin alleviates apoptosis.

**FIGURE 10:**
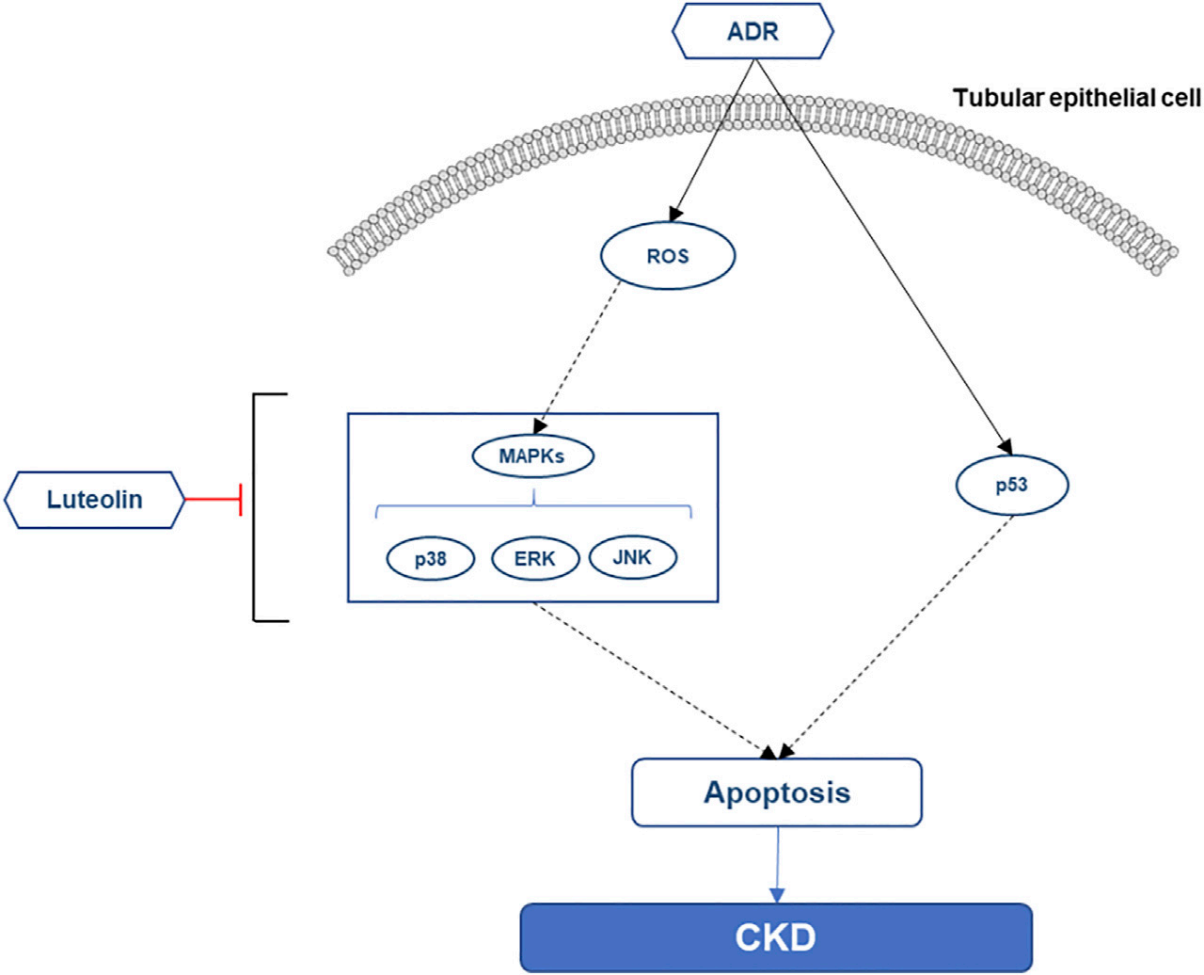
Possible mechanism of luteolin alleviating the apoptosis of renal tubular epithelial cells.

Finally, we summarized the possible mechanism of luteolin to reduce the apoptosis of renal tubular cells (Figure 10). Additionally, the key pathway maps of MAPK and P53 were also retrieved from KEGG database to provide reference for further in-depth exploration of the mechanism (Supplementary Figures S4A,B).

## Discussion

Network pharmacological analyses have demonstrated that the main active components of *P. frutescens* are luteolin, β-sitosterol, rosmarinic acid, caffeic acid, and β-carotene. These components can play a therapeutic role in kidney disease by regulating immunity, inflammation, and metabolism (Fuyuno et al., 2018; Huang et al., 2018; Wei et al., 2018). Through their intersection with renal injury-related genes, 108 targets were presently identified. Analysis of these related targets revealed that the core nodes in the PPI network included AKT1, TP53, IL6, TNF, MAPK1, JUN, VEGFA, EGFR, CASP3, CCND1, MYC, RELA, MMP9, IL2, and PTGS2. These are involved in the immune response, inflammation, cell proliferation, and apoptosis. They are closely related to the development and prognosis of CKD. The findings suggest that the above proteins may be the main targets of *P. frutescens* in the treatment of CKD. At the same time, the analysis of the main active component-target network and PPI network revealed the complex and close relationships between each component and target, which are connected to each other in a variety of ways.

GO enrichment analysis revealed that these targets are involved in the composition of various CCs, which regulate the activity of various kinases, nuclear receptors, transcription factors, and the binding capacity of cytokine receptors, chemokine receptors, catecholamines, phosphatases, and proteases. The CCs may have therapeutic roles in CKD stage 3–5 by affecting BPs, such as regulation of transcription and apoptosis, protein phosphorylation, inflammatory response, aging, and cell proliferation.

The enrichment analysis of the KEGG signaling pathway showed that *P. frutescens* slows the progression of CKD by regulating the apoptosis, TCR, PI3K-AKT, HIF-1, TLR, and TNF signaling pathways. Apoptosis disorders are closely associated with CKD. Apoptosis signaling pathways mainly include MAPK, NF-κB, PI3K-AKT, and others, which are closely related to CKD and jointly induce activation of downstream caspase families. These pathways induce abnormal apoptosis of renal TECs and vascular endothelial cells and mediate the continuous progression of the disease. As an important part of cellular immunity, T cells mediate the immune response of the body, promote the release of inflammatory factors and aggravate the inflammatory response. Moreover, the TCR signaling pathway can also regulate Treg differentiation, which plays a crucial role in maintaining homeostasis and physiological function (Li and Rudensky, 2016). The PI3K/AKT pathway is closely related to cell growth and proliferation. Regulation of this pathway can affect renal mesangial cell proliferation and extracellular matrix secretion (Son et al., 2020). Chronic hypoxia is an important pathological factor in CKD. Under hypoxia, the expression of HIF-1α increases, which activates the transcription of downstream factors, such as VEGF and erythropoietin, and improves the hypoxic status of the body (Mudaliar et al., 2013). The inflammatory response is key to the deterioration of CKD. TLR is a ligand-activated membrane-bound receptor that mainly causes an inflammatory response by activating NF-κB. TLR also initiates a downstream signaling cascade reaction, which leads to renal TEC injury and aggravates renal function (Miyata et al., 2013). TNF-α is the most important cytokine in the TNF family. It can initiate downstream biological reactions, such as inflammation and apoptosis, by binding to TNF receptor 1 on the cell surface, including participation in the induction of the caspase family mediated apoptosis and activation of NF-κB, p38, ERK, and other signaling pathways to promote downstream inflammatory responses (Akdis et al., 2016).

It should be noted that, the targets of these active components overlap and cross in multiple pathways and biological functions. For example, the apoptosis signaling pathway in enrichment analysis contained 14 key targets, and the HIF-1 signaling pathway also had 15 targets. Conversely, many targets are involved in multiple biological functions and signaling pathways too. AKT, a key node in the network model, is involved in 45 of all 254 enriched BPs. And it also contributes to 59 enriched signaling pathways, such as apoptosis pathway, HIF-1 pathway, and PI3K-Akt pathway. Recently, research has found that the frequently used herb pairs tend to have shorter distances compared to random herb pairs, and the center distance determined at the ingredient level improves the discrimination of top-frequent herb pairs from random herb pairs (Wang et al., 2021). It provides us with an effective methodological reference, whether in exploring the rules of classic TCM formulas, herb combination, or pharmacological analysis. In supplementary materials Supplementary Table S1, we also enumerated some network topology characteristics of the active components and targets, such as Betweenness Centrality and Closeness Centrality. But we have not yet conducted an in-depth and comprehensive discussion on it. This network pharmacology modeling provides a new perspective and method for our follow-up research, and help us to better explore the space of herbal combinations more effectively.

Molecular docking was used to verify the binding ability of the main active components and their corresponding therapeutic targets. The binding ability was good, which further reflected the therapeutic effect of the main active components of *P. frutescens*.

Luteolin, rosmarinic acid, caffeic acid, perilla aldehyde and (+)-catechin were detected by HPLC to construct PFAE fingerprint. These components were also coincident with the aforementioned components obtained from TCMSP database and network pharmacological analysis.

Finally, we selected luteolin, a key component of *P. frutescens*, to verify the mechanism of kidney protection *in vitro*. Accumulating evidence has suggested that luteolin exhibits anti-oxidation, anti-inflammatory, and anti-fibrotic effects (Alekhya et al., 2019). Previous studies have reported that luteolin can reduce renal ischemia-reperfusion injury, lipopolysaccharide or cisplatin induced acute kidney injury, and angiotensin-induced renal injury (Domitrović et al., 2013; Xin et al., 2016; Hong et al., 2017; Liu et al., 2021), but there are few studies on its pharmacological mechanism in CKD. In our study, we found that luteolin can significantly mitigate apoptosis induced by ADR and modulated the phosphorylation of AKT and p53. Meanwhile, the detection of oxidative stress and the key pathway closely related to apoptosis revealed that luteolin can inhibit oxidative stress and down-regulate MAPK pathway. These results suggest that the MAPK and p53 pathways could be the key mechanisms by which luteolin exerts its anti-apoptotic pharmacological effects.

In conclusion, in this study, we clarified the multi-component, multi-target, multi-pathway effects of *P. frutescens* by network pharmacological analysis. Luteolin, the critical component of *P. frutescens*, was identified by network pharmacology and HPLC. *In vitro* experiments, we verified that both luteolin and *P. frutescens* can effectively reduce renal tubular injury, and investigated the mechanism by which luteolin alleviates kidney injury. Luteolin can reduce ADR-induced apoptosis of TECs. Anti-oxidation, down-regulate MAPKs phosphorylation and inhibition of the p53 pathway could be the key mechanisms in it. Our findings revealed that luteolin may be the essential component of *P. frutescens* for treating CKD stage 3–5, which provides a basis and direction for further mechanism research. At the same time, the results also enriched and improved the pharmacological research of *P. frutescens*. It will also help us perform in-depth research on the herb and related formula in the future. However, in this study, we did not verify specific interrelationships between these pathways and apoptosis. We are planning experiments to clarify the specific mechanisms by adding positive control agents, gene transfection and *in vivo* experiments. In addition, we mainly focus on a single component called luteolin. The interaction between various components and targets constitutes the clinical therapeutic effect of TCM, and it is also the feature and advantage of TCM treatment. Recently, researchers have continuously constructed a variety of new network pharmacology models and platforms, thus providing effective strategies for us to better explore herb pairs and formulas (Zhou et al., 2016; Bu et al., 2020; Wang et al., 2021). Therefore, more in-depth studies on drug-drug interactions and combinations might be needed in the future, so as to more comprehensively elucidate the clinical efficacy mechanism of TCM.

## Data Availability

The datasets presented in this study can be found in online repositories. The names of the repository/repositories and accession number(s) can be found in the article/Supplementary Material.

## Abbreviations

ADR, adriamycin; BP, biological process; CASP3, caspase 3; CC, cellular component; CCND1, cyclin D1; CKD, chronic kidney disease; CRF, chronic renal failure; DL, drug-like; EGFR, epidermal growth factor receptor; EPO, erythropoietin; ERK 1/2, extracellular signal-regulated kinase 1/2; HIF-1α, hypoxia inducible factor-1α; HPLC, high performance liquid chromatograph; IL6, interleukin 6; JNK, c-Jun N-terminal kinase; MAPK, mitogen-activated protein kinase; MF, molecular function; MMP9, matrix metalloproteinase 9; NAC, N-acetyl-L-cysteine; NCOA2, nuclear receptor coactivator 2; NF-κB, nuclear factor-κ-gene binding; NRK-52E, normal rat tubular proximal epithelial cell; OB, oral bioavailability; PARP1, poly (ADP-ribose) polymerase 1; PFAE, P. frutescens aqueous extracts; PI3K, phosphoinositide 3-kinase; PPI, protein-protein interaction; PTGS 2, prostaglandin-endoperoxide synthase 2; ROS, reactive oxygen species; TCM, traditional Chinese medicine; TCR, T cell receptor; TEC, tubular epithelial cell; TLR, Toll-like receptor; TNF, tumor necrosis factor; TP53, tumor protein 53; VEGF, vascular endothelial growth factor.
