# Supplementary figures and images for: Exploring the Critical Components and Therapeutic Mechanisms of Perilla frutescens L. in the Treatment of Chronic Kidney Disease via Network Pharmacology
Source: Front Pharmacol. 2021 Nov 26;12:717744. doi: 10.3389/fphar.2021.717744 (PMC8662752; doi:10.3389/fphar.2021.717744)

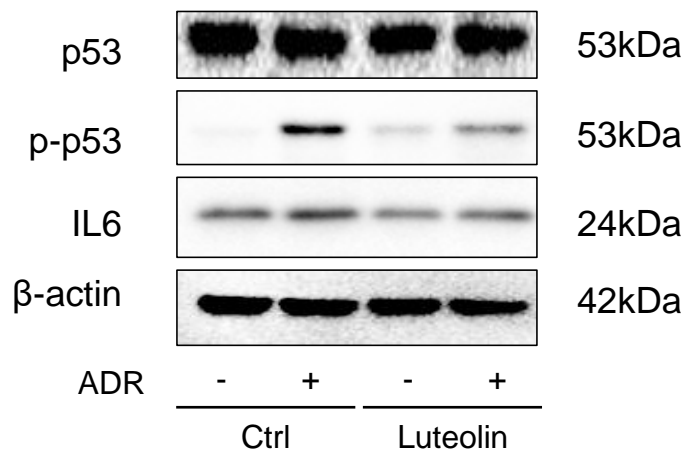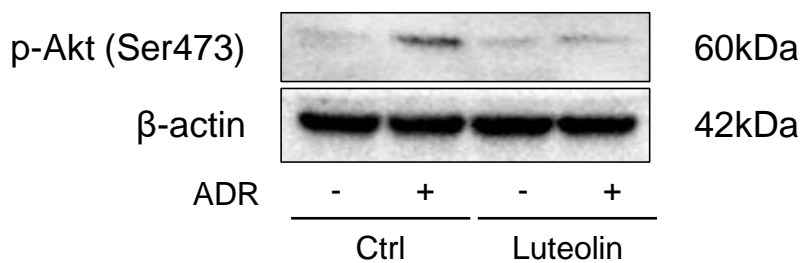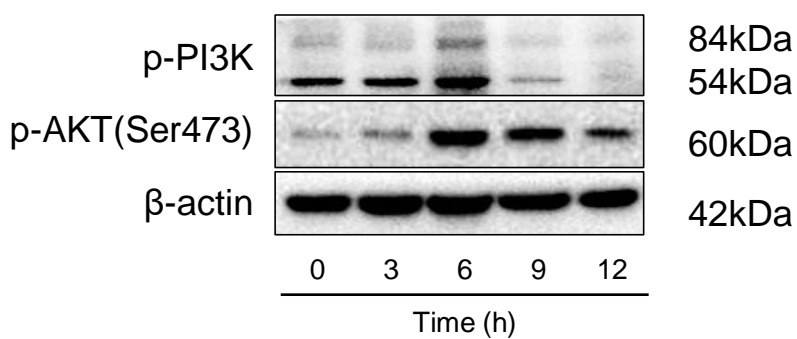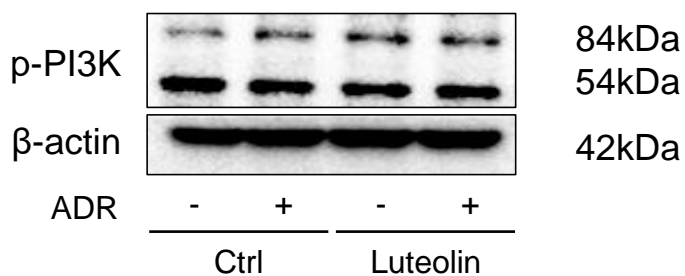

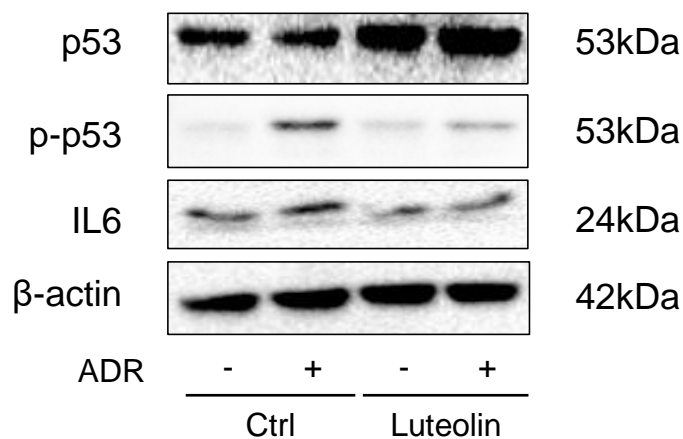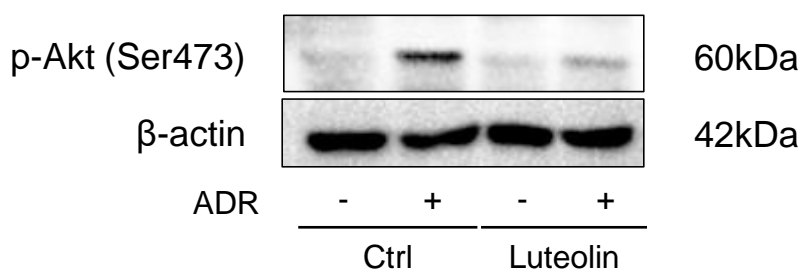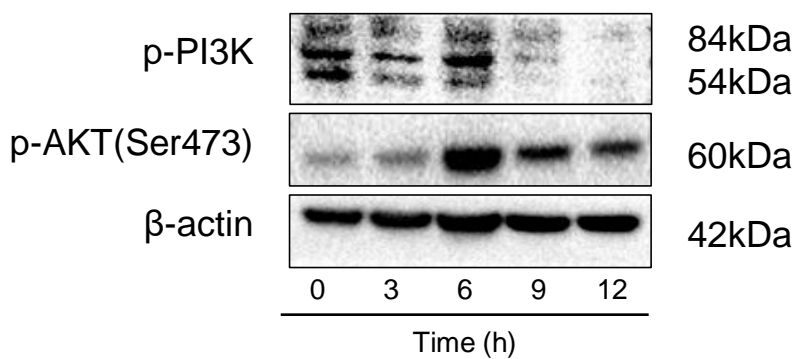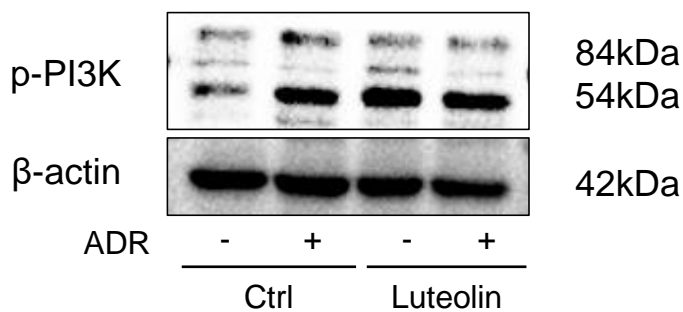

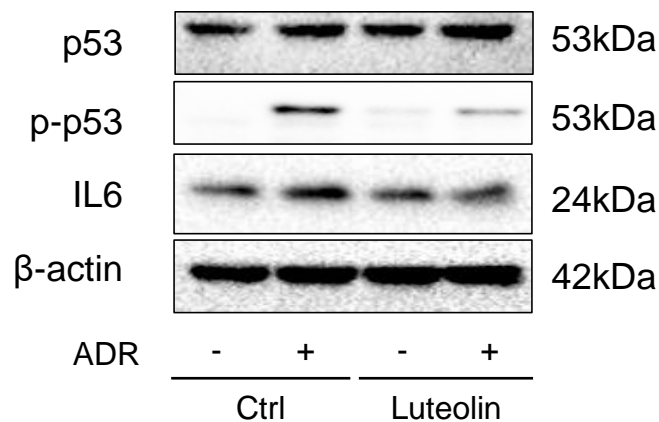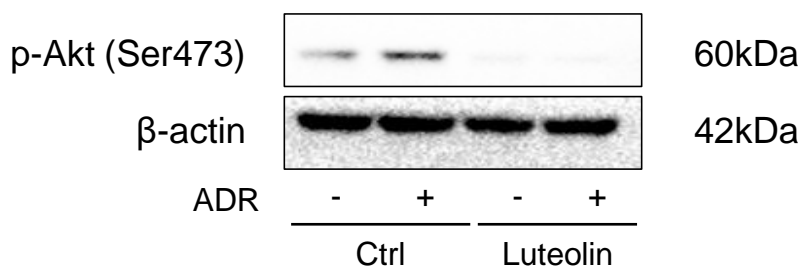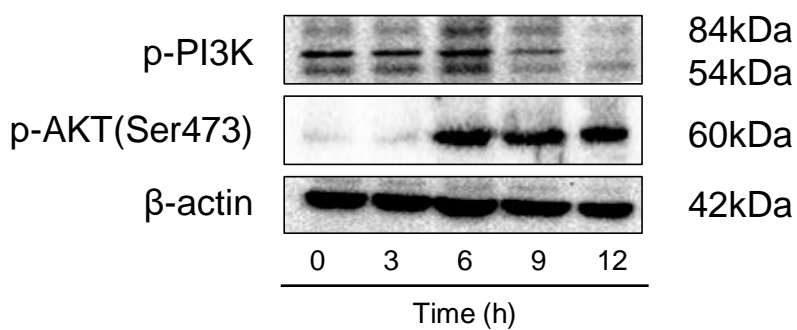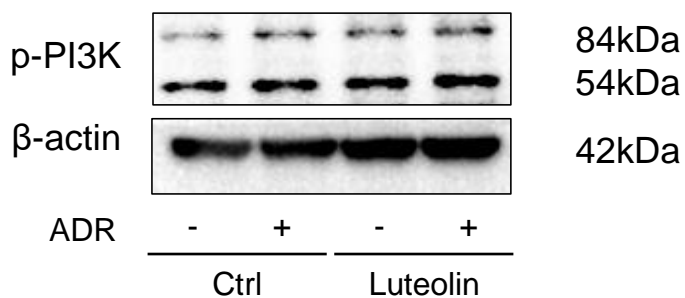

Supplement: Supplementary file 1 [file DataSheet2.PDF]

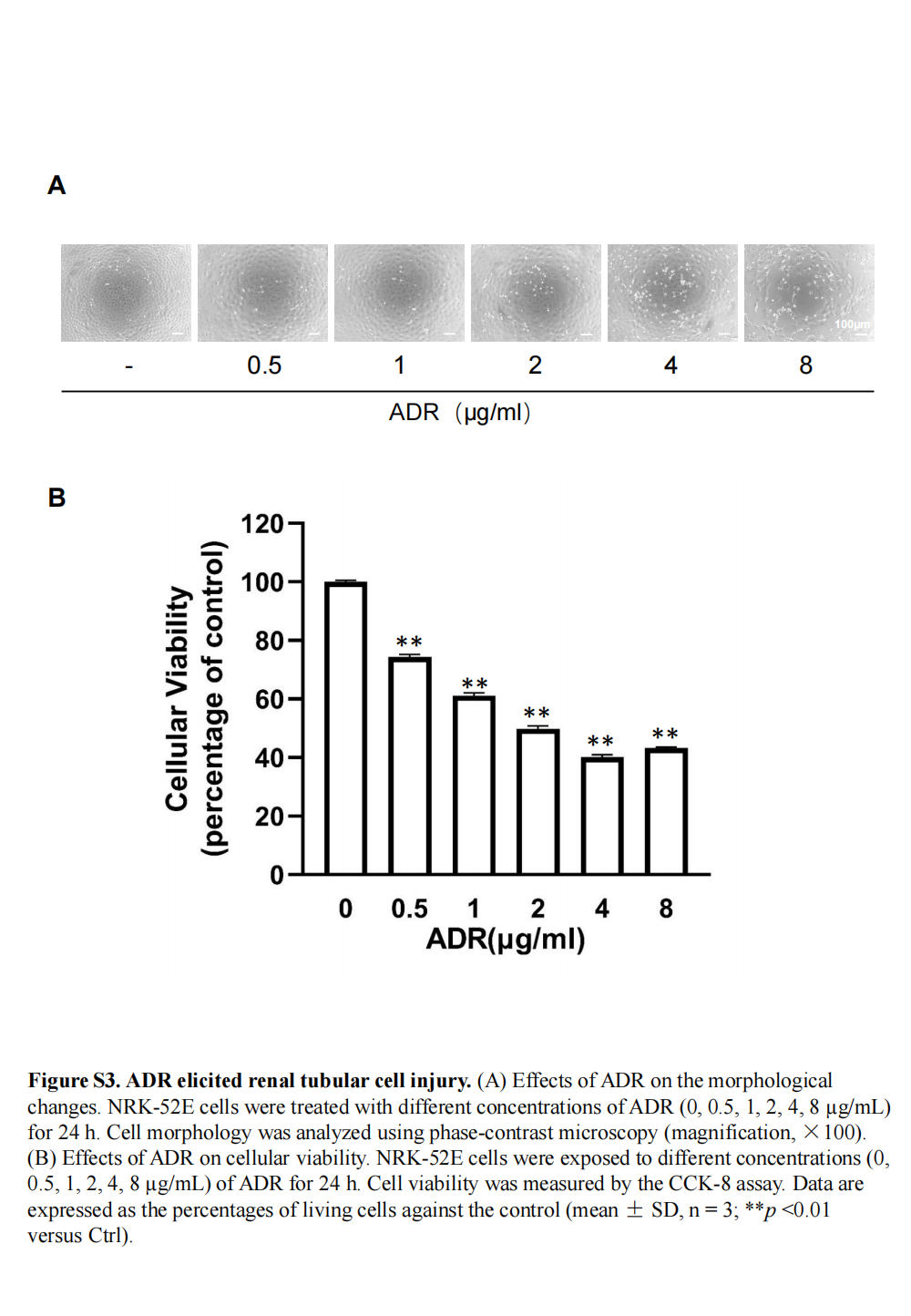

Supplement: Supplementary file 3 [file Image3.TIF]

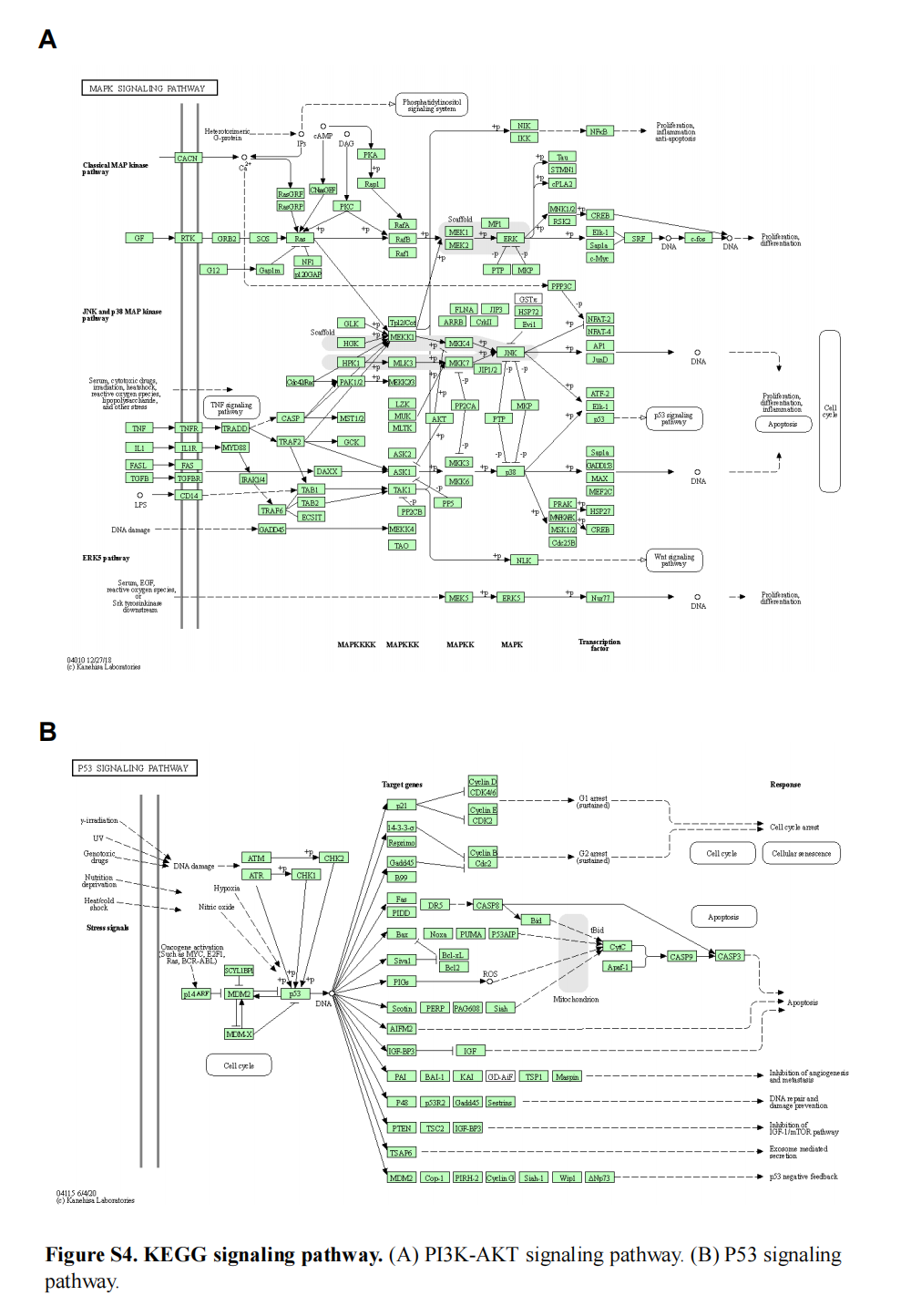

Supplement: Supplementary file 4 [file Image4.TIF]

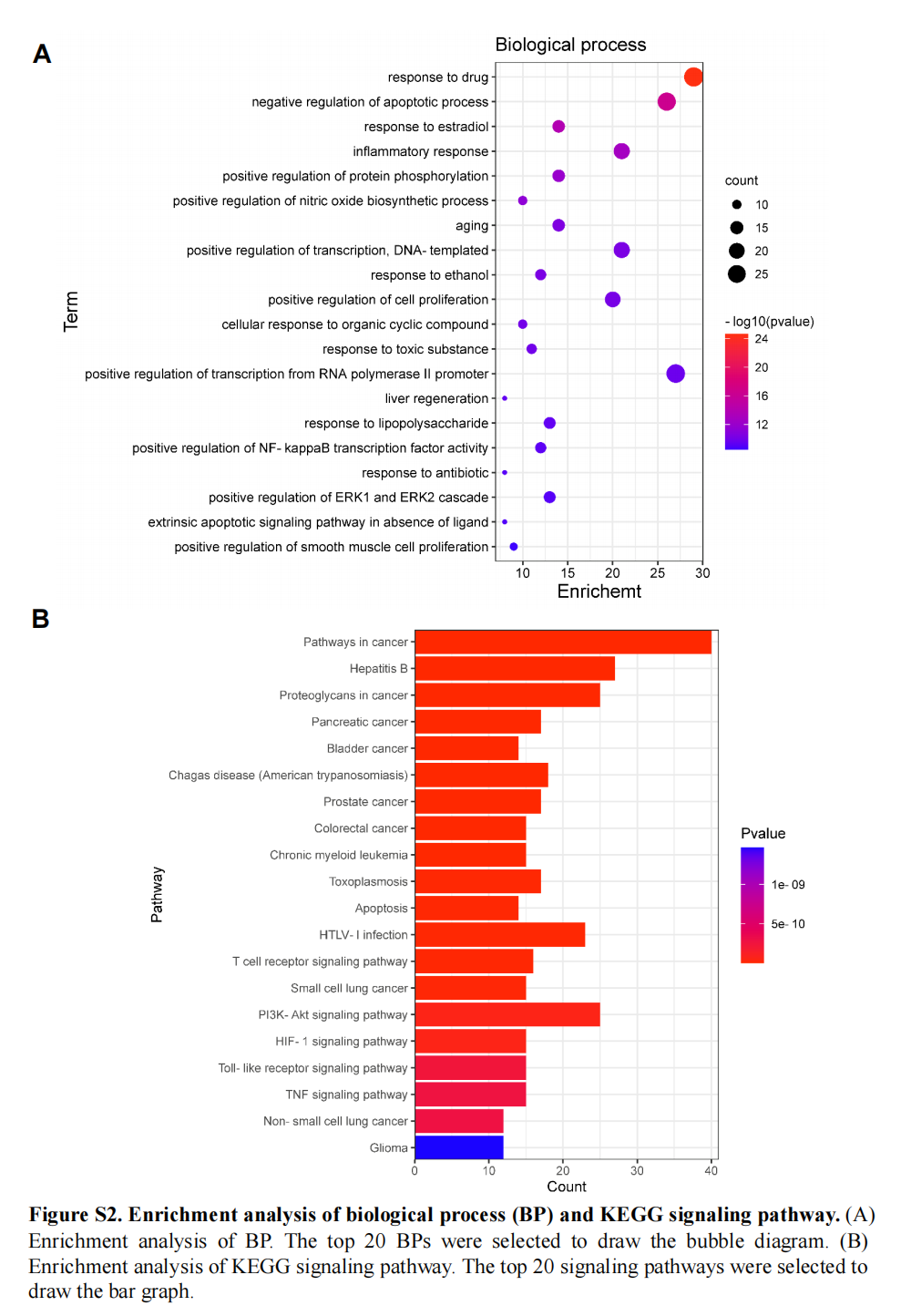

Supplement: Supplementary file 5 [file Image2.TIF]

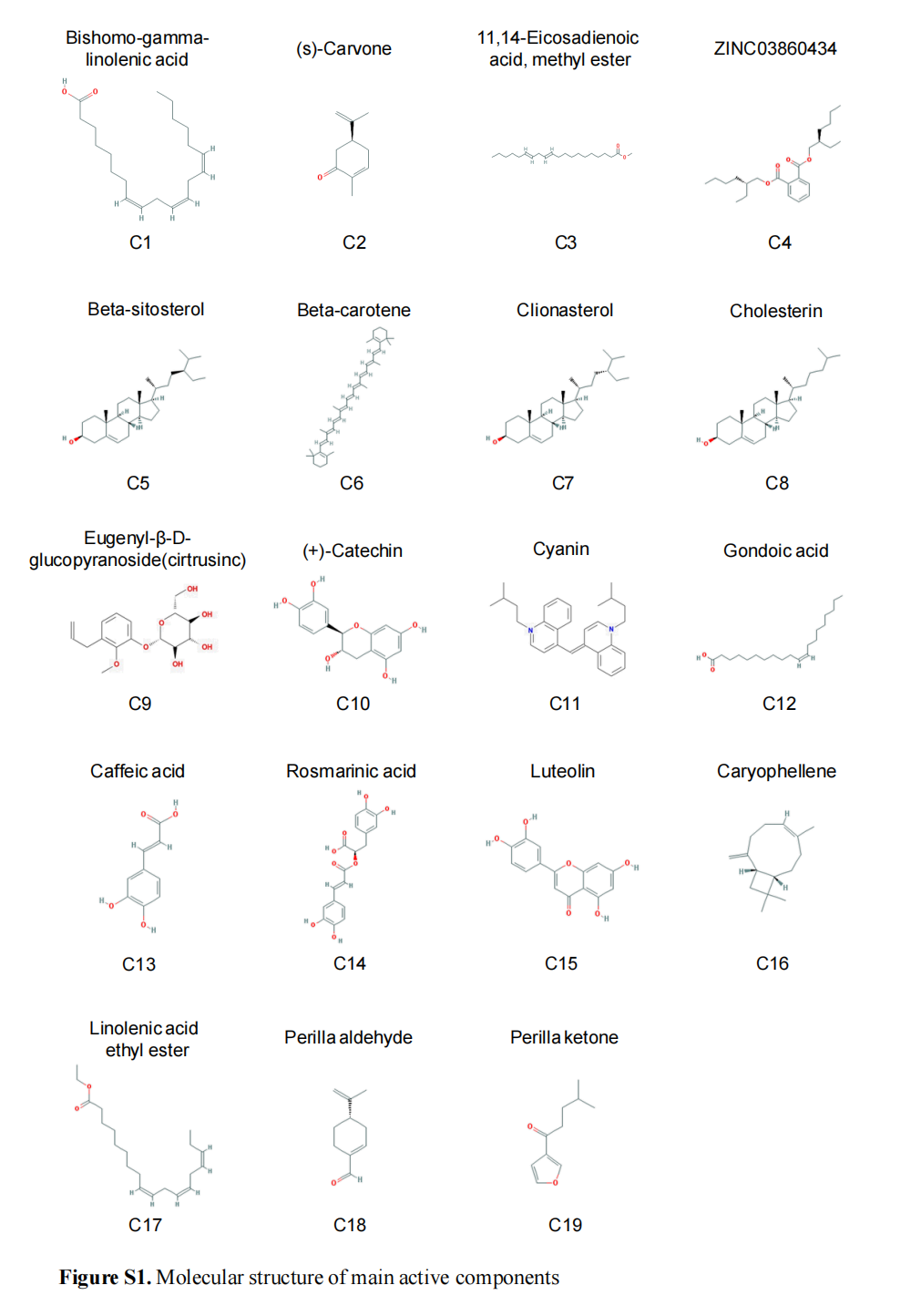

Supplement: Supplementary file 6 [file Image1.TIF]

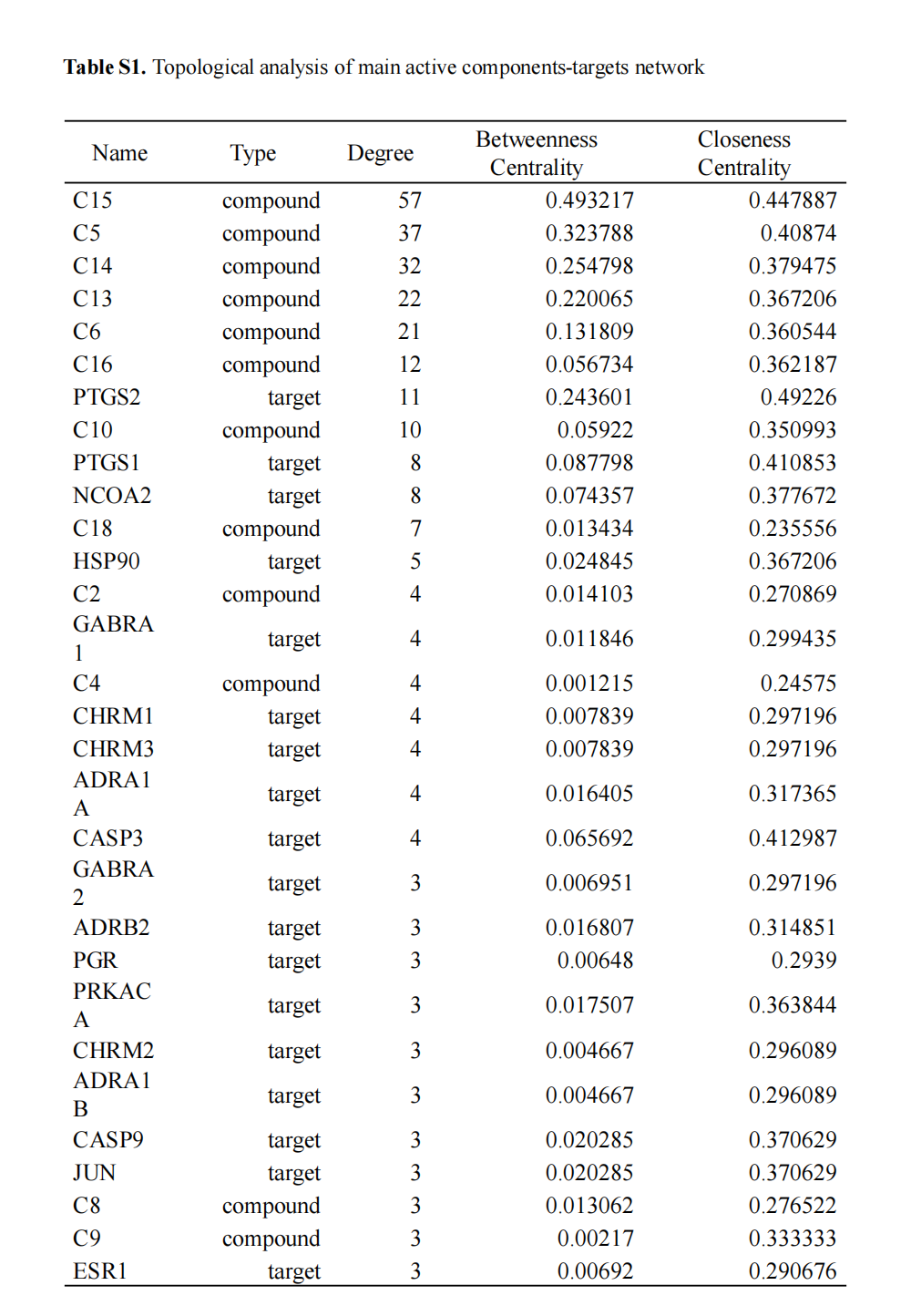

Supplement: Supplementary file 8 [file Image5.TIF]
