# Supplementary material for: Exploring the Critical Components and Therapeutic Mechanisms of Perilla frutescens L. in the Treatment of Chronic Kidney Disease via Network Pharmacology
Source: Front Pharmacol. 2021 Nov 26;12:717744. doi: 10.3389/fphar.2021.717744 (PMC8662752; doi:10.3389/fphar.2021.717744)
Supplement: Supplementary file 7 [file DataSheet1.PDF]

Experimental Data

Morphology and viability

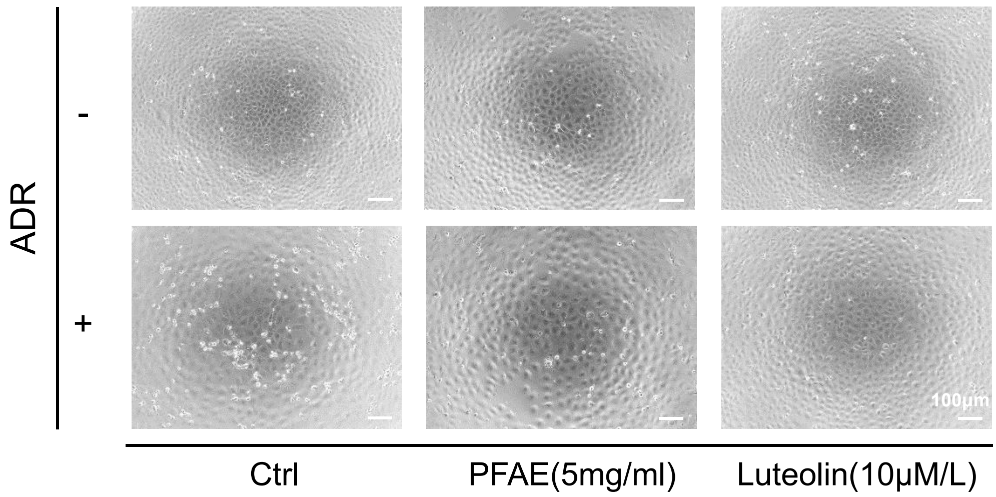

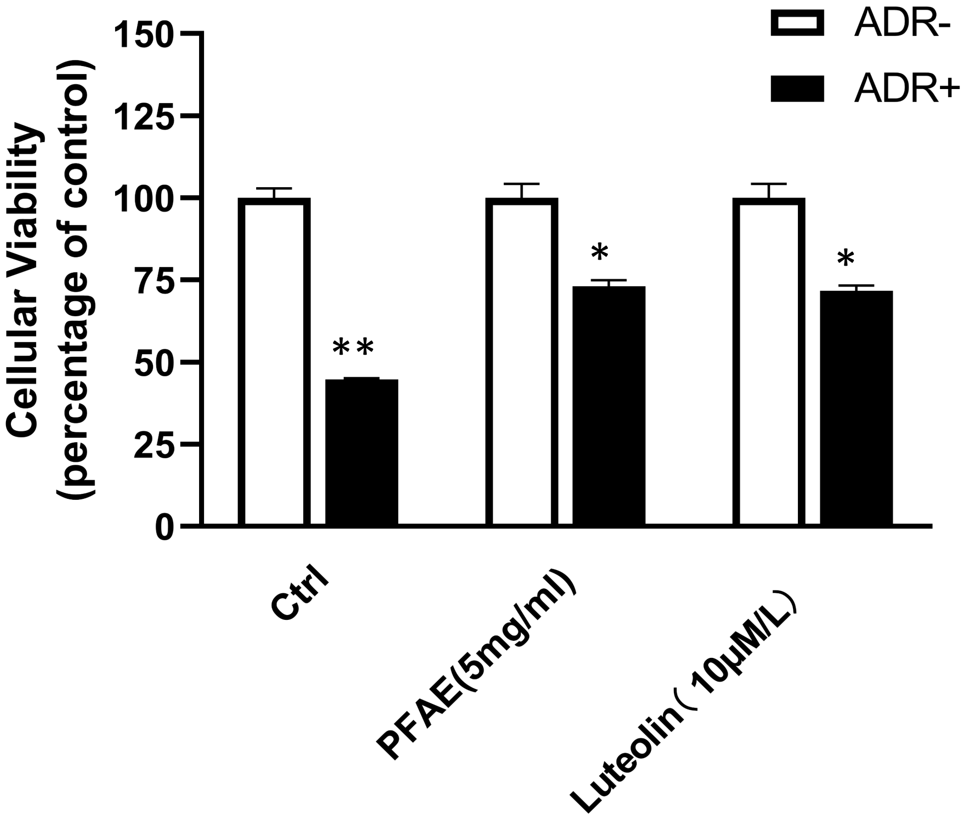

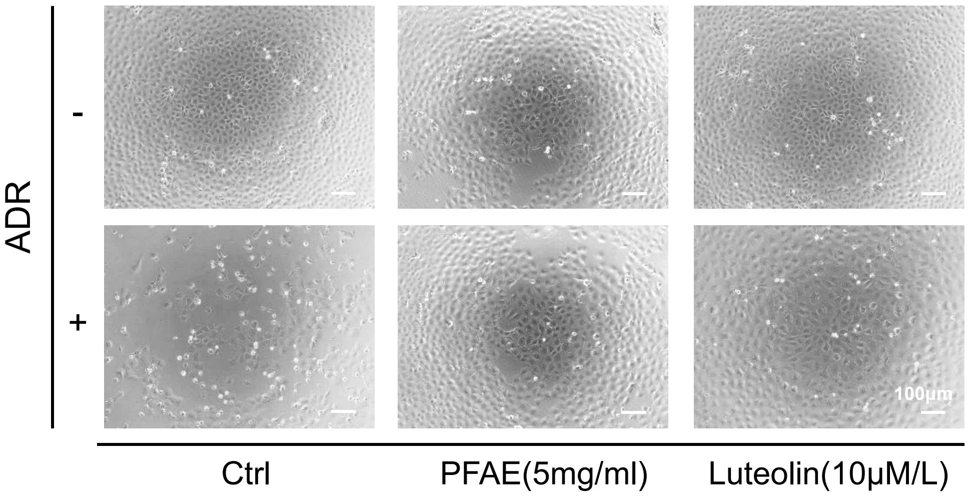
ADR
-
+
100μm
Ctrl
PFAE(5mg/ml)
Luteolin(10μM/L)

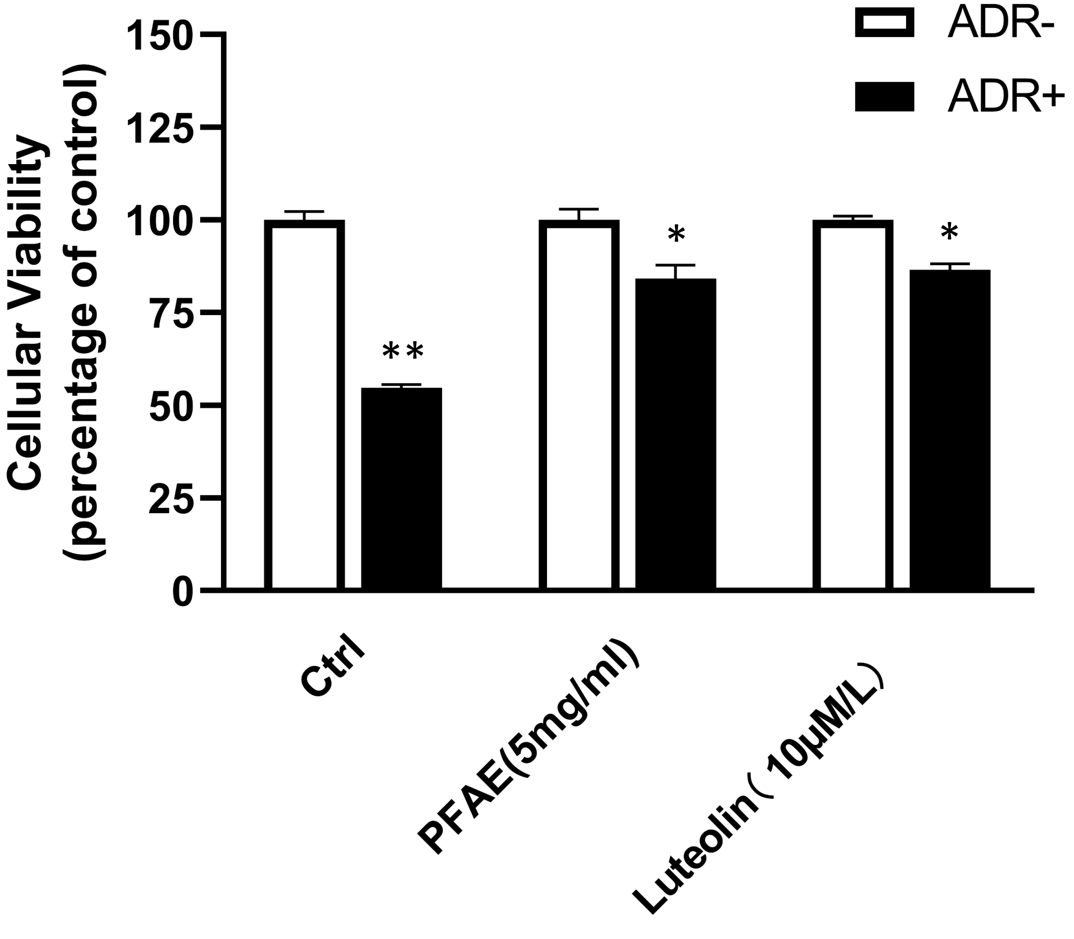
Cellular Viability
(percentage of control)
150
125
100
75
50
25
0
ADR-
ADR+
**
*
*
Ctrl
PFAE(5mg/ml)
Luteolin（10μM/L）

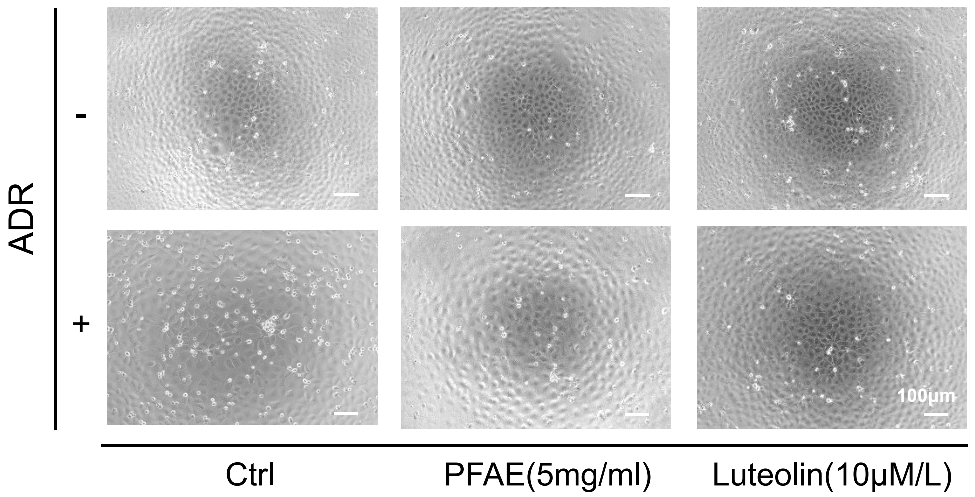
ADR
-
+
100μm
Ctrl
PFAE(5mg/ml)
Luteolin(10μM/L)

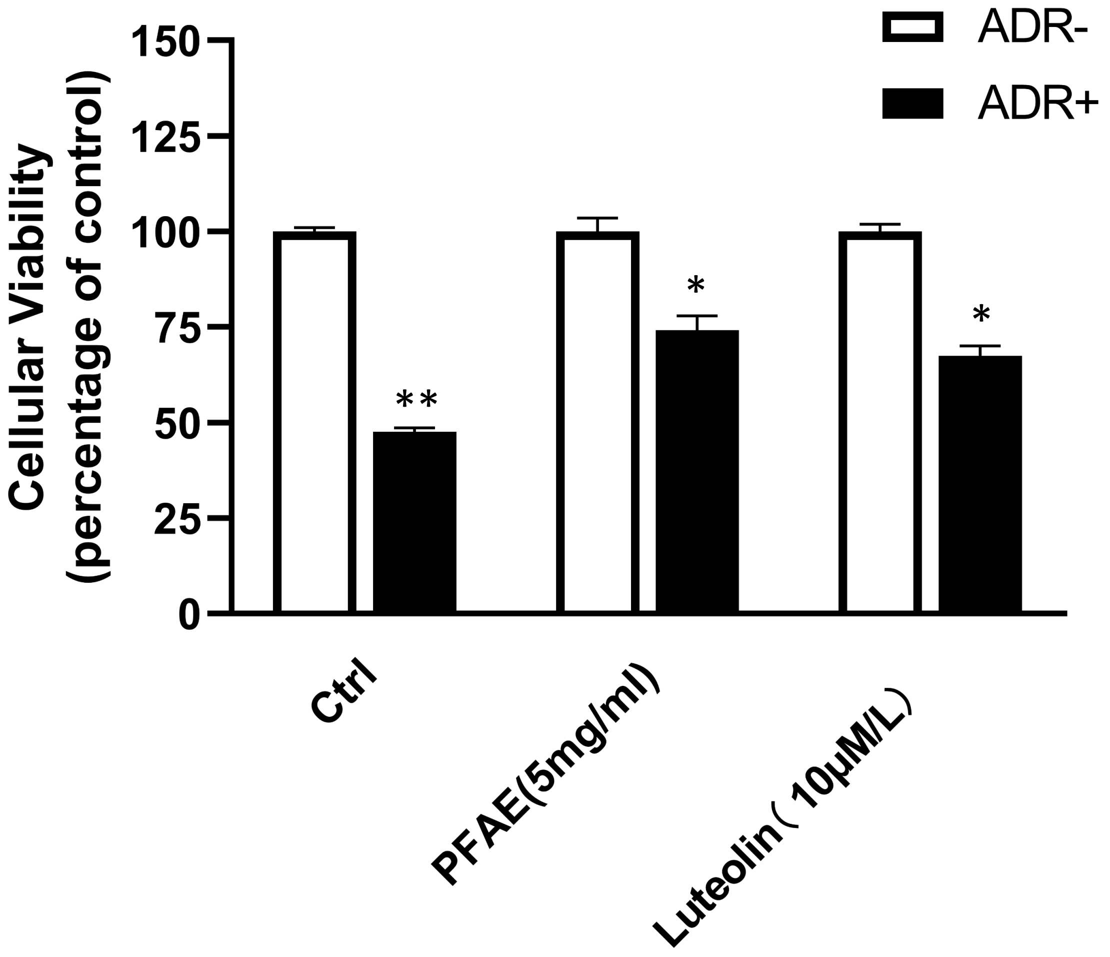
ADR-
ADR+
Cellular Viability (percentage of control)
150
125
100
75
50
25
0
**
*
*
Ctrl
PFAE(5mg/ml)
Luteolin（10μM/L）

ROS fluorescence staining

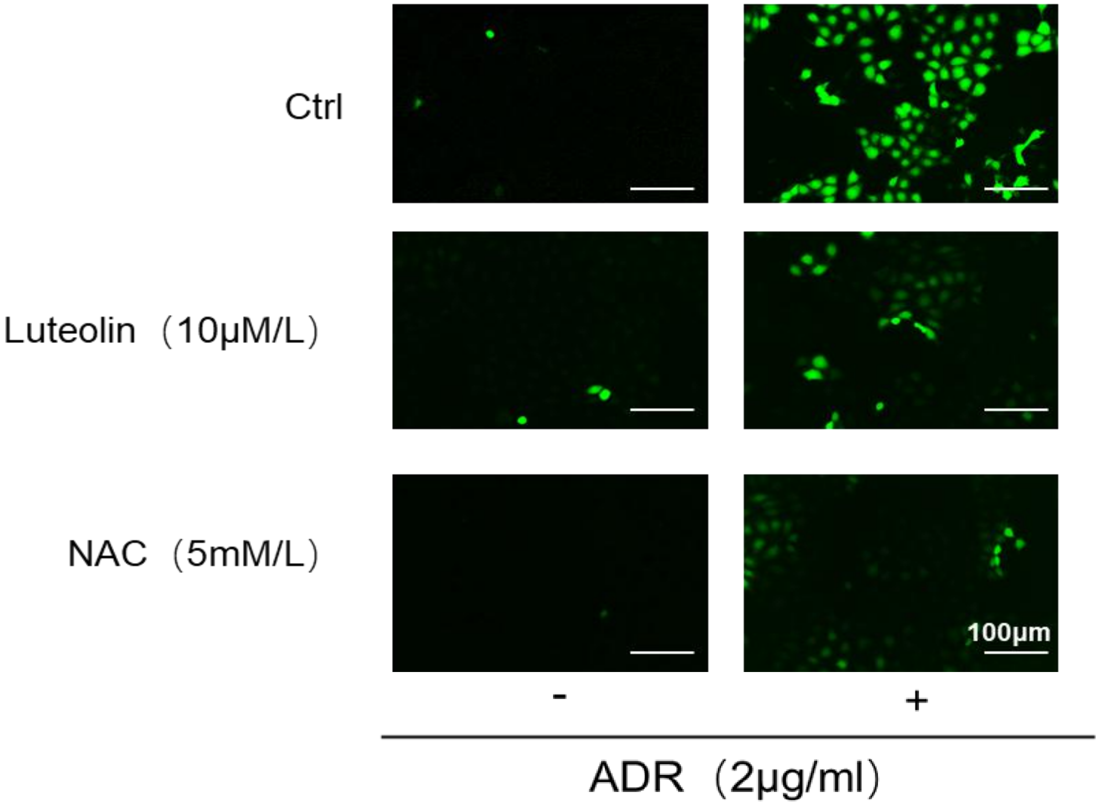

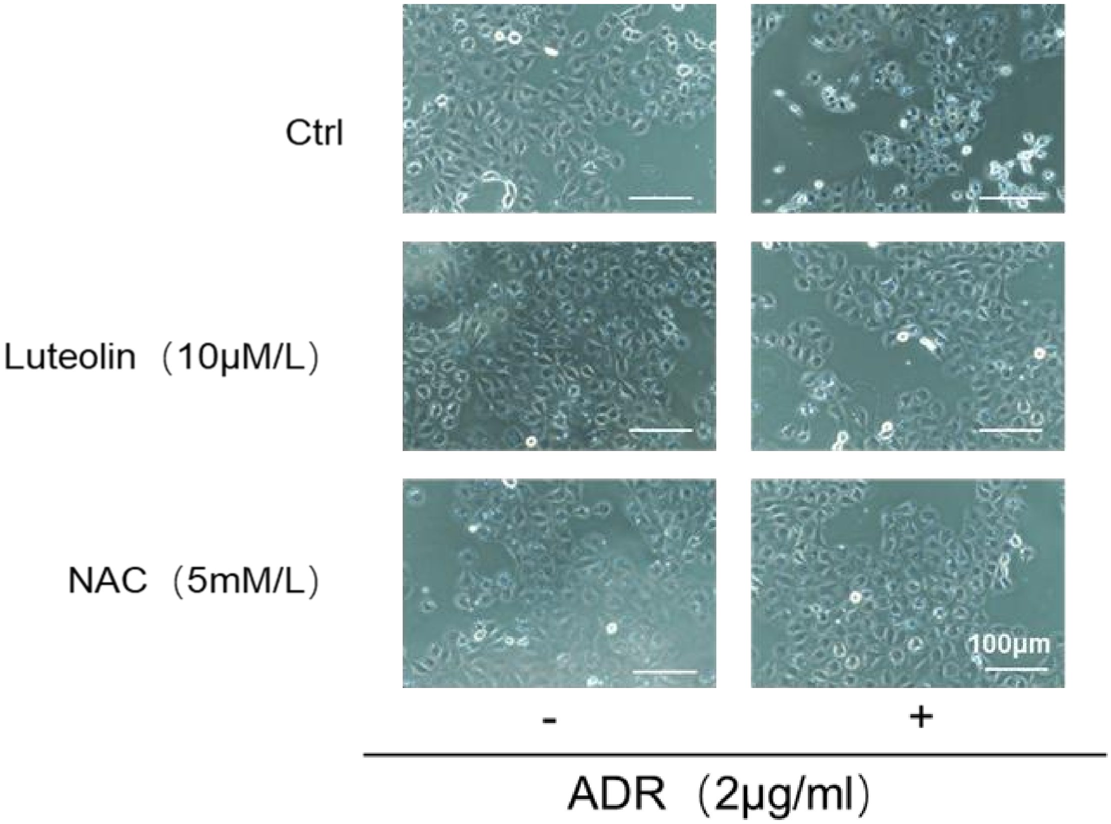

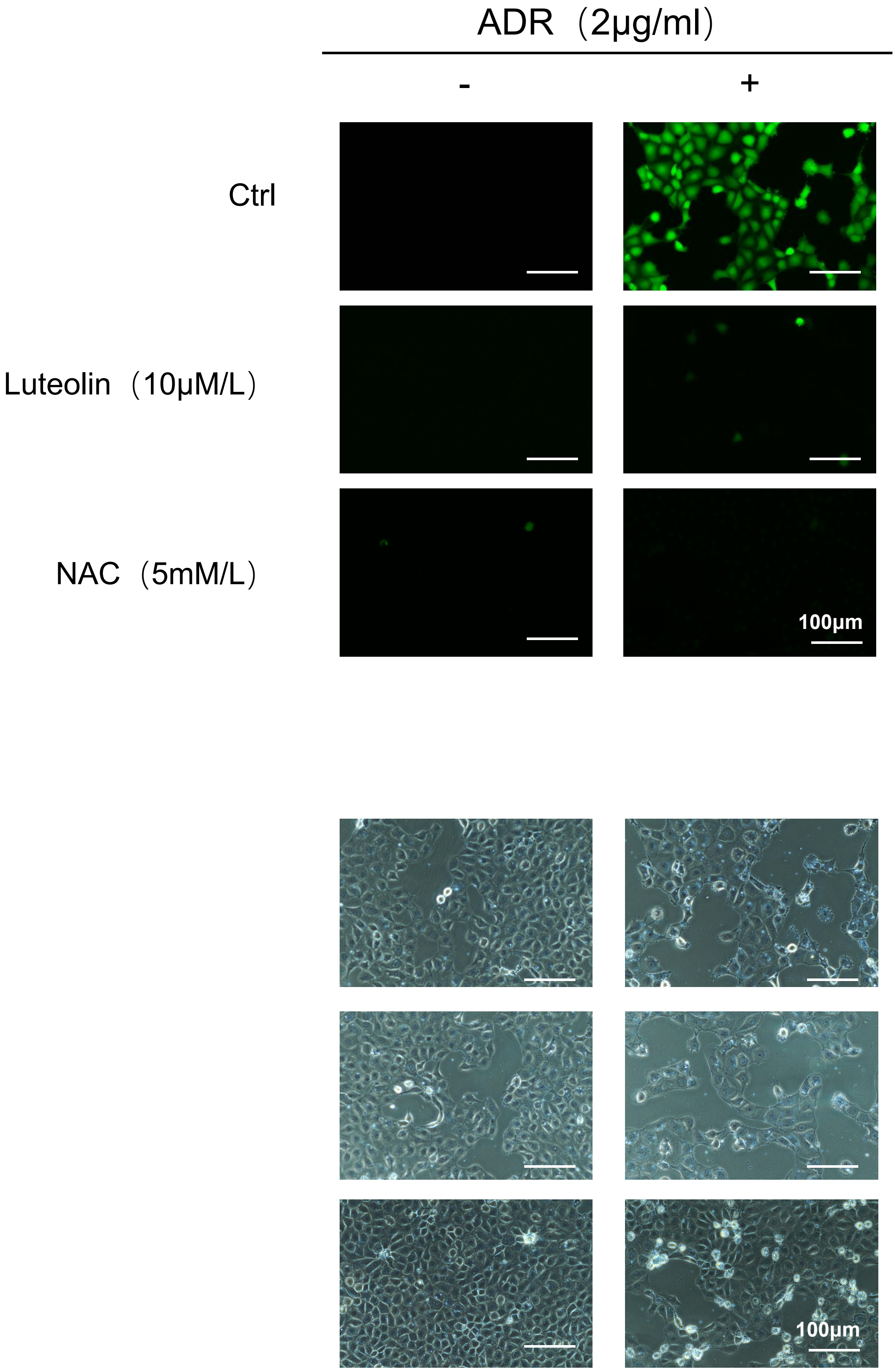
ADR（2μg/ml）
-
+
Ctrl
Luteolin（10μM/L）
NAC（5mM/L）
100μm
100μm

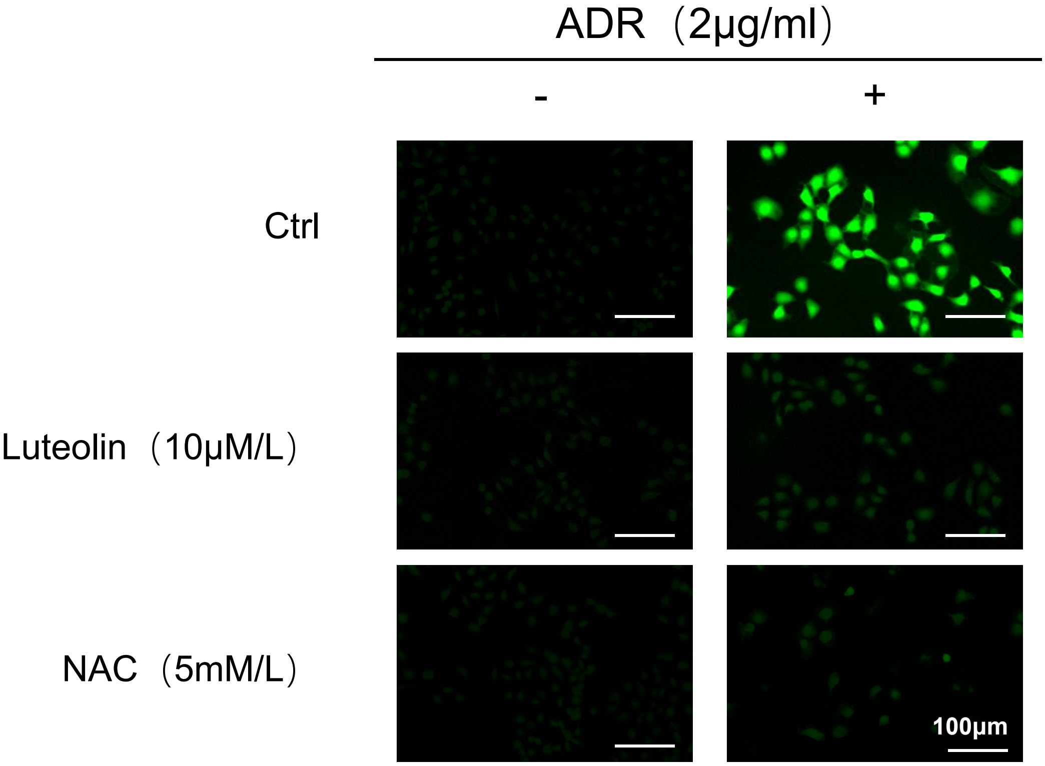
ADR（2μg/ml）
-
+
Ctrl
Luteolin（10μM/L）
NAC（5mM/L）
100μm

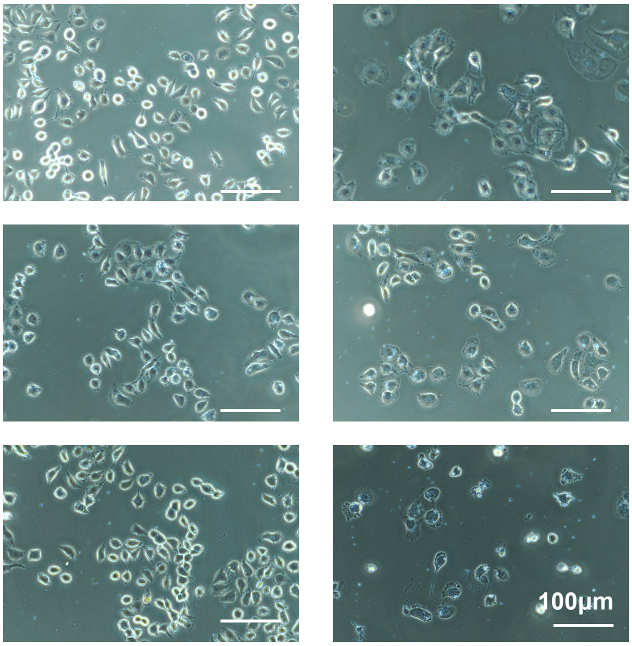
100μm

TUNEL+DAPI staining

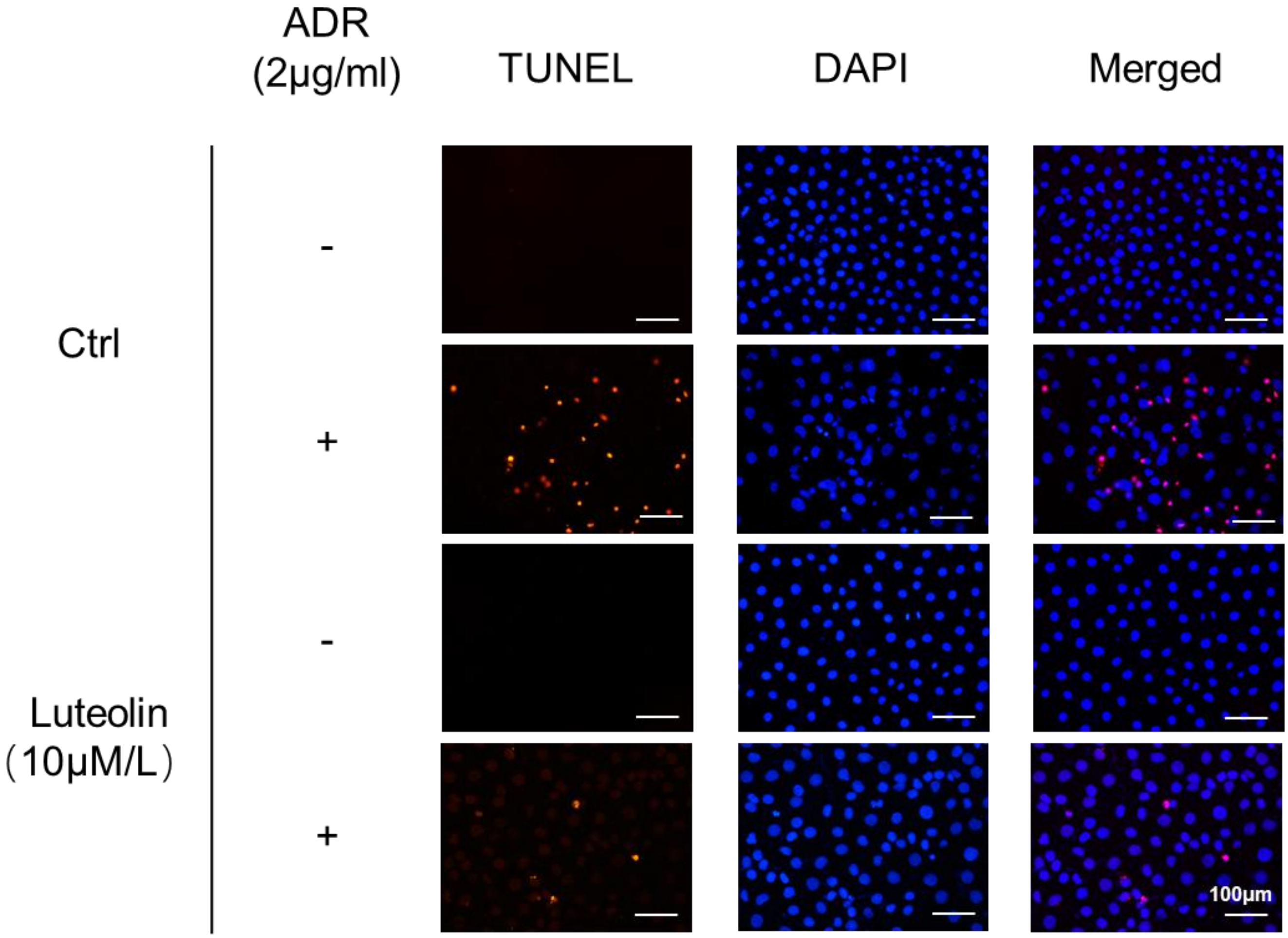

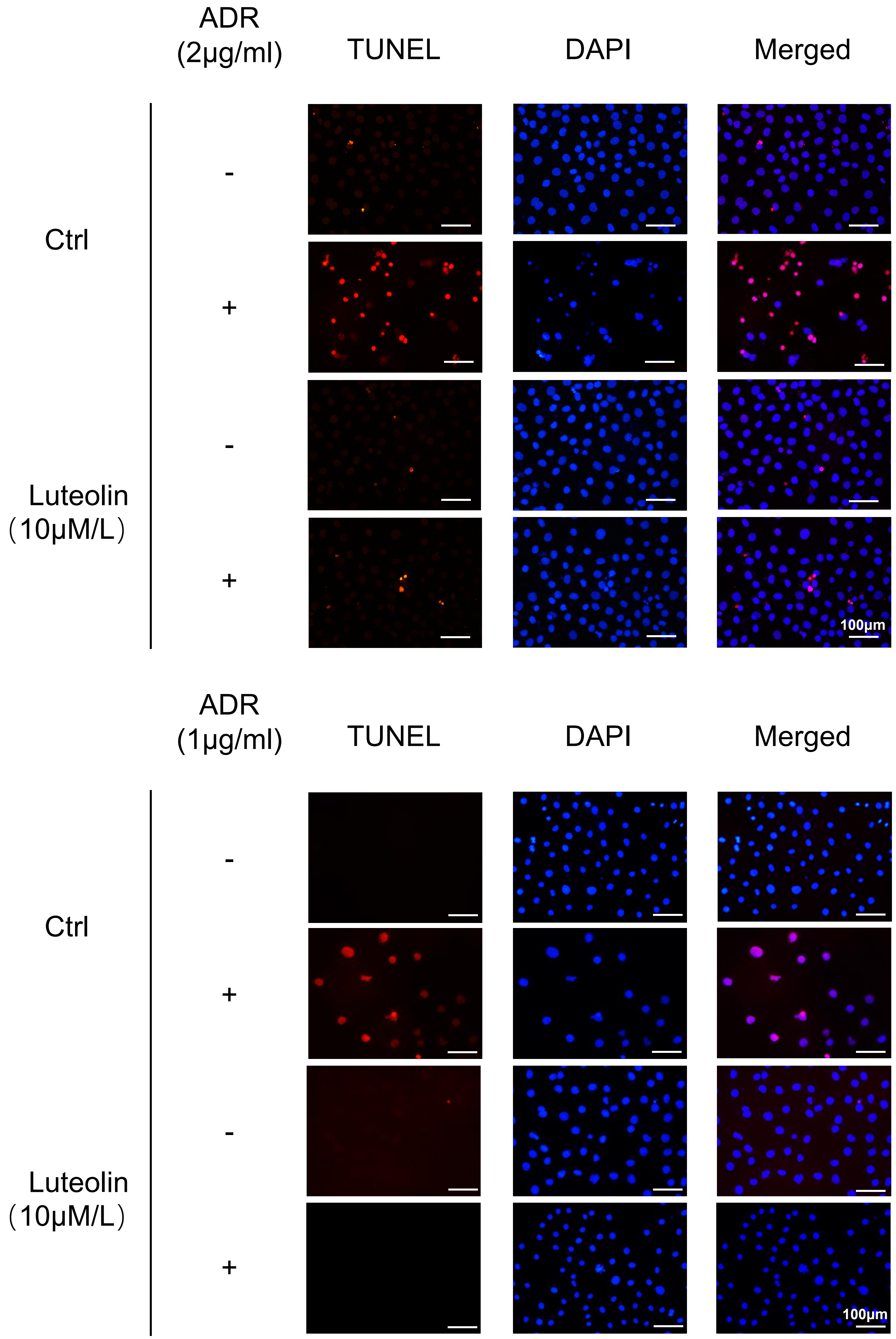
ADR (2μg/ml)
TUNEL
DAPI
Merged
Ctrl
Luteolin (10μM/L)
-
+
-
+
100μm
ADR (1μg/ml)
TUNEL
DAPI
Merged
Ctrl
Luteolin (10μM/L)
-
+
-
+
100μm

Flow cytometry

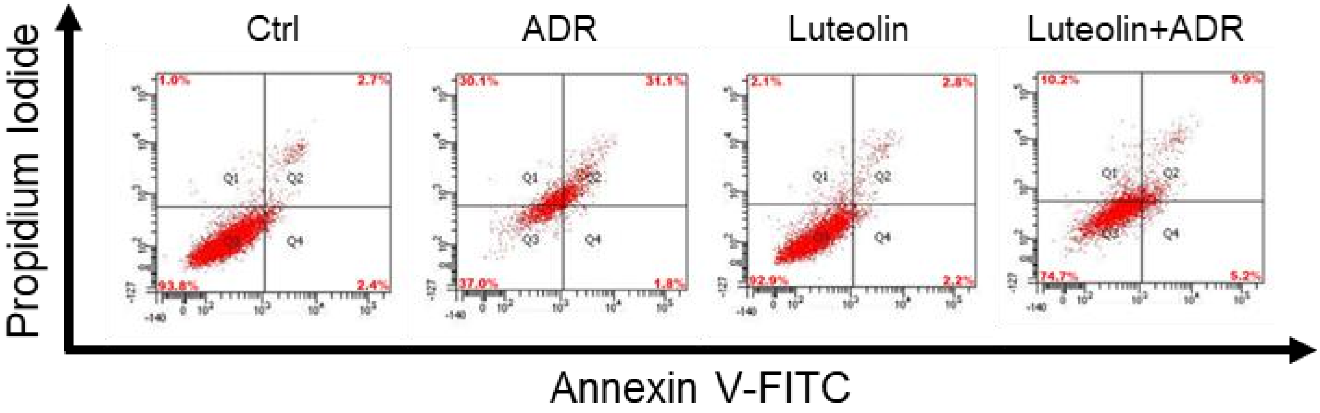

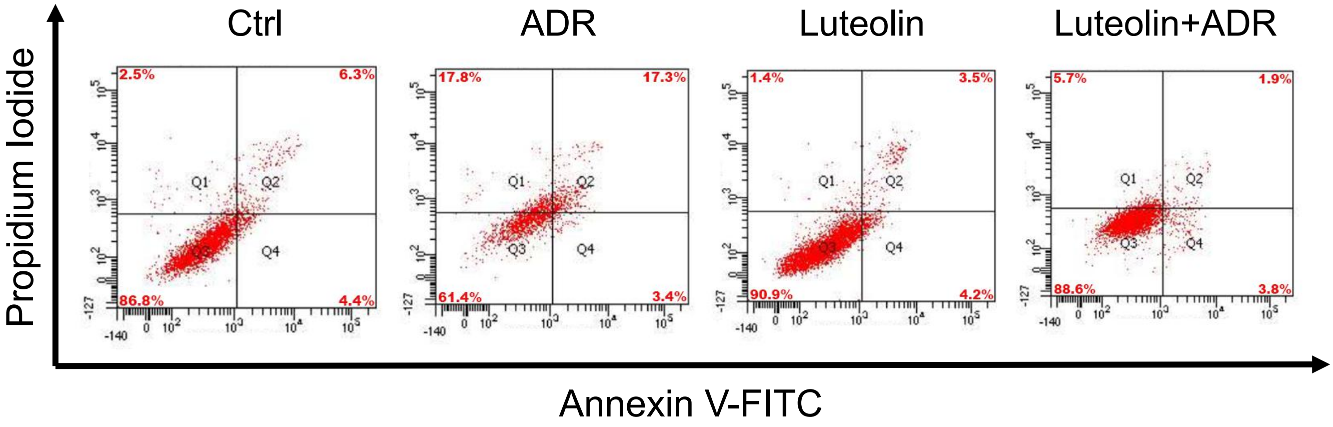

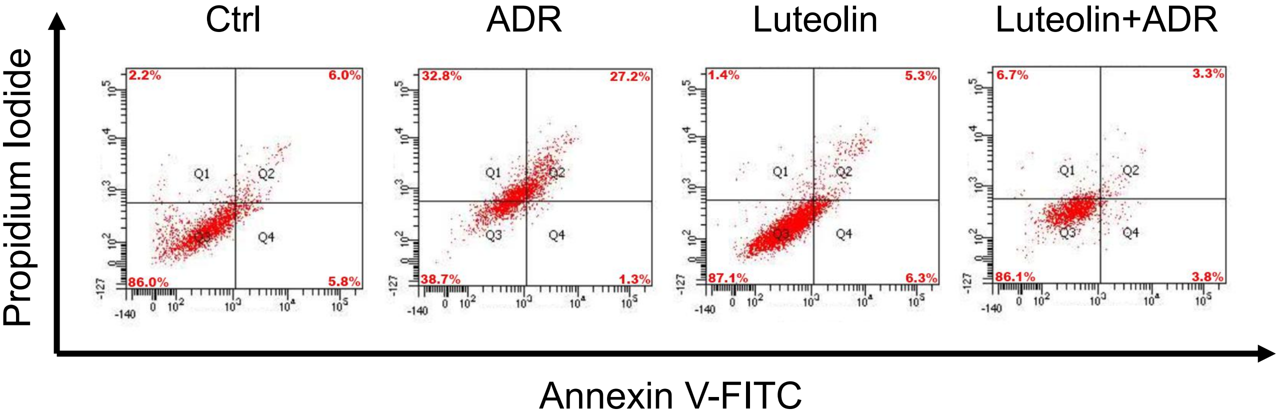

Western Blot

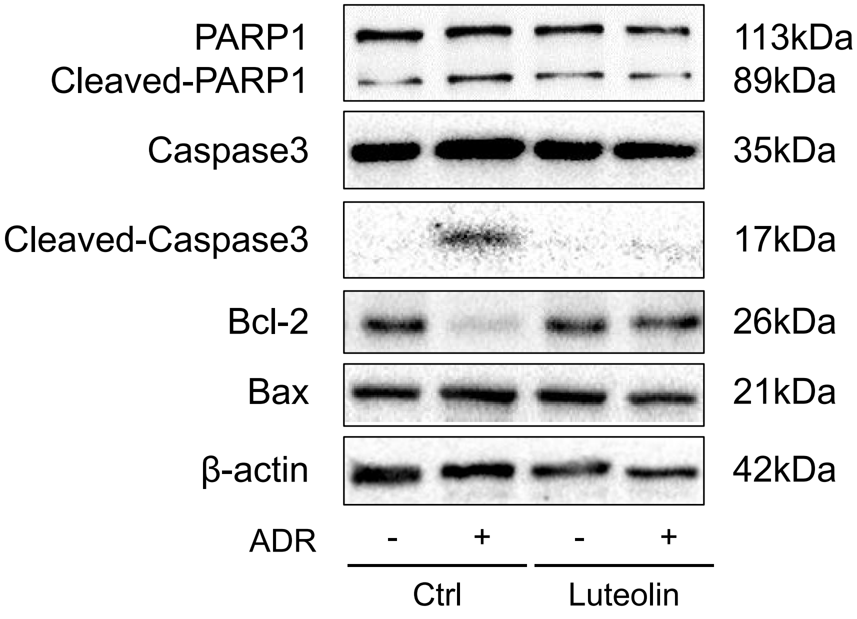

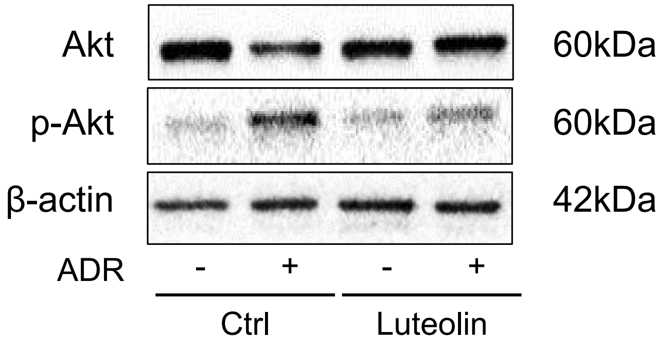

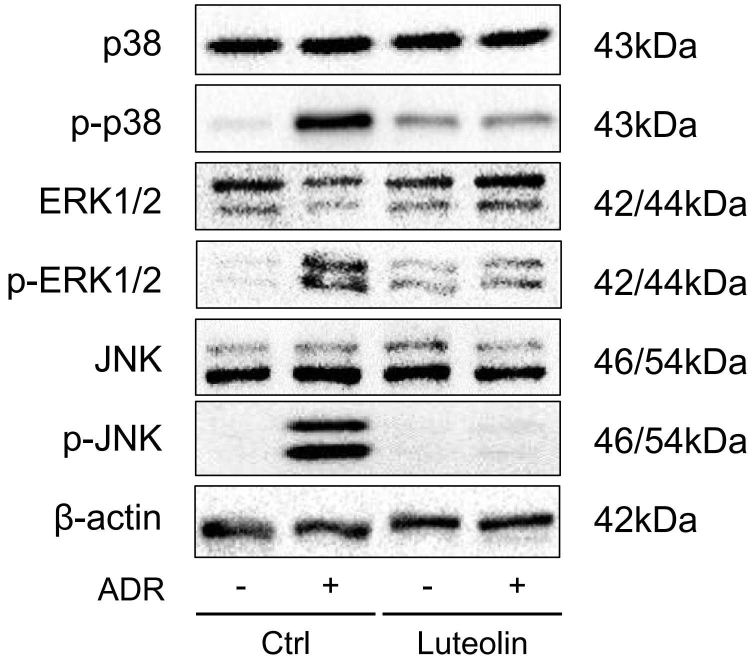

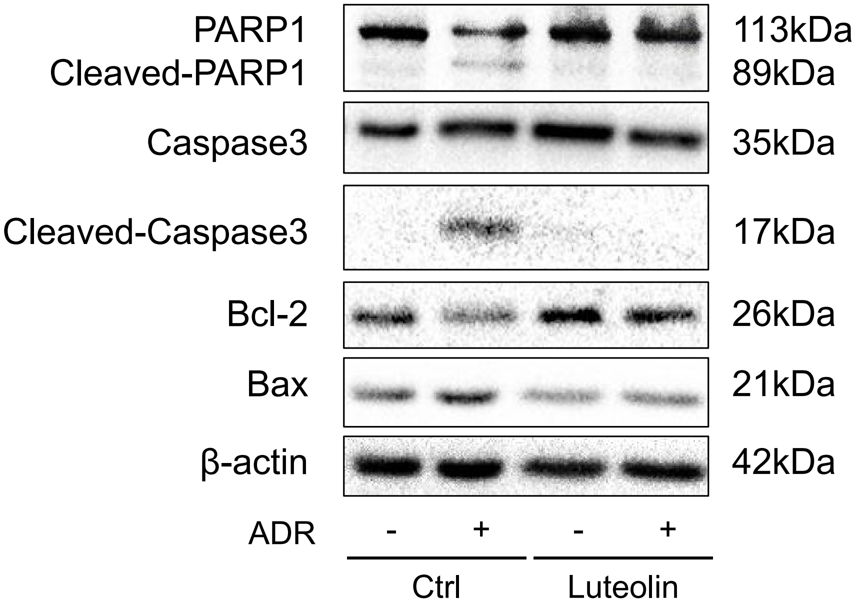
PARP1
Cleaved-PARP1
Caspase3
Cleaved-Caspase3
Bcl-2
Bax
β-actin
113kDa
89kDa
35kDa
17kDa
26kDa
21kDa
42kDa
ADR
- + - +
Ctrl
Luteolin

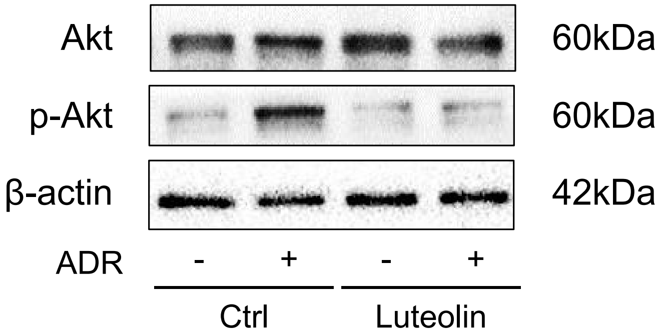
Akt
p-Akt
β-actin
60kDa
60kDa
42kDa
ADR
- + - +
Ctrl
Luteolin

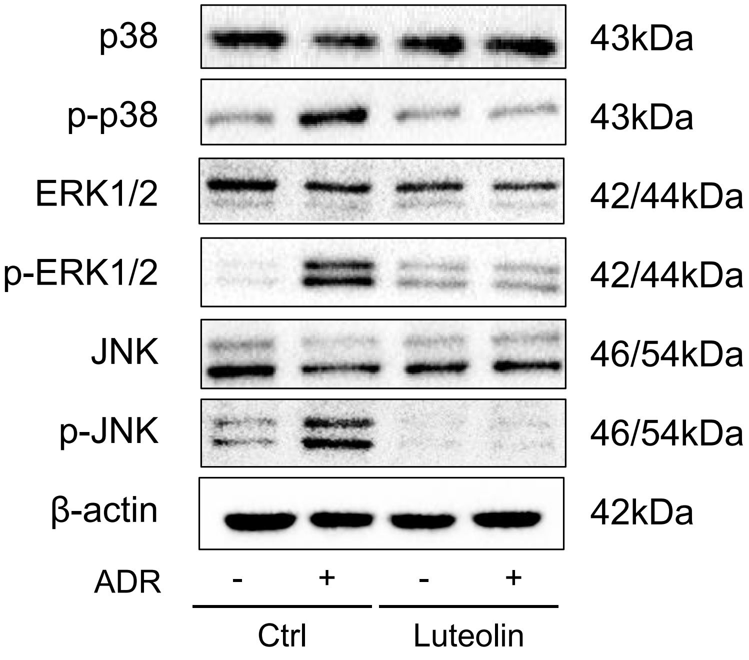
p38
p-p38
ERK1/2
p-ERK1/2
JNK
p-JNK
β-actin
43kDa
43kDa
42/44kDa
42/44kDa
46/54kDa
46/54kDa
42kDa
ADR
- + - +
Ctrl
Luteolin

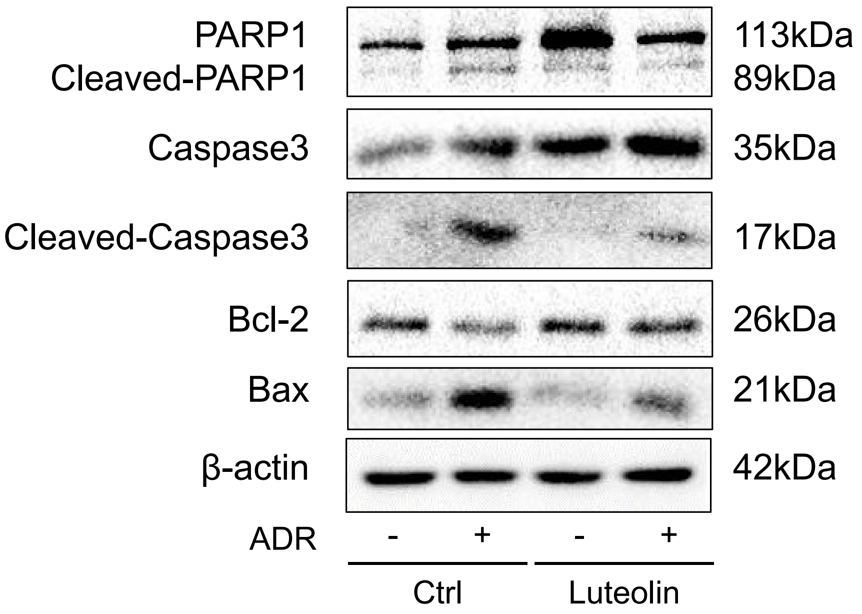
PARP1
Cleaved-PARP1
Caspase3
Cleaved-Caspase3
Bcl-2
Bax
β-actin
113kDa
89kDa
35kDa
17kDa
26kDa
21kDa
42kDa
ADR - + - +
Ctrl
Luteolin

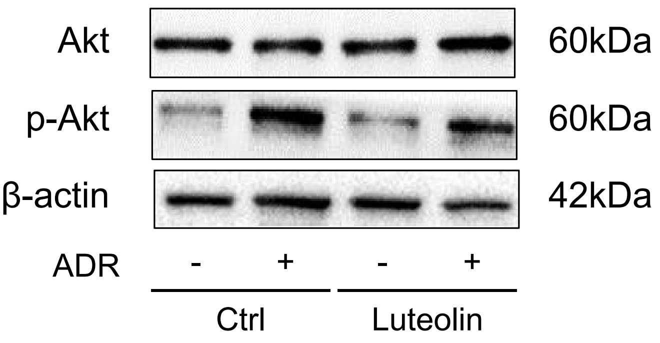
Akt
p-Akt
β-actin
60kDa
60kDa
42kDa
ADR - + - +
Ctrl
Luteolin

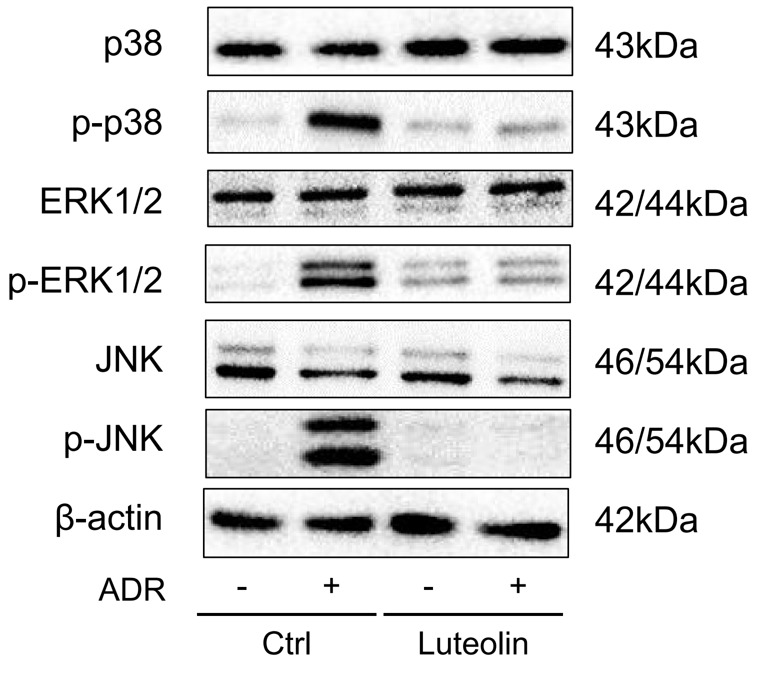
p38
p-p38
ERK1/2
p-ERK1/2
JNK
p-JNK
β-actin
43kDa
43kDa
42/44kDa
42/44kDa
46/54kDa
46/54kDa
42kDa
ADR - + - +
Ctrl
Luteolin
